# *AtHSPR* is involved in GA- and light intensity-mediated control of flowering time and seed set in Arabidopsis

**DOI:** 10.1093/jxb/eraa128

**Published:** 2020-03-10

**Authors:** Tao Yang, Yan Sun, Yongli Wang, Lina Zhou, Mengya Chen, Zhiyuan Bian, Yuke Lian, Lijuan Xuan, Guoqiang Yuan, Xinyu Wang, Chongying Wang

**Affiliations:** 1 Ministry of Education Key Laboratory of Cell Activities and Stress Adaptations, School of Life Sciences, Lanzhou University, Lanzhou, China; 2 Trinity College Dublin, Ireland

**Keywords:** *Arabidopsis thaliana*, *AtHSPR*, flowering time, seed set, gibberellin, light intensity

## Abstract

Flowering is a dynamic and synchronized process, the timing of which is finely tuned by various environmental signals. A T-DNA insertion mutant in Arabidopsis *HEAT SHOCK PROTEIN-RELATED* (*AtHSPR*) exhibited late-flowering phenotypes under both long-day (LD) and short-day (SD) conditions compared to the wild-type, while over-expression of *AtHSPR* promoted flowering. Exogenous application of gibberellin (GA) partially rescued the late-flowering mutant phenotype under both LD and SD conditions, suggesting that *AtHSPR* is involved in GA biosynthesis and/or the GA signaling that promotes flowering. Under SD or low-light conditions, the *Athspr* mutant exhibited late flowering together with reduced pollen viability and seed set, defective phenotypes that were partially rescued by GA treatment. qRT-PCR assays confirmed that GA biosynthetic genes were down-regulated, that GA catabolic genes were up-regulated, and that the levels of bioactive GA and its intermediates were decreased in *Athspr* under both SD and low-light/LD, further suggesting that *AtHSPR* could be involved in the GA pathway under SD and low-light conditions. Furthermore, AtHSPR interacted *in vitro* with OFP1 and KNAT5, which are transcriptional repressors of *GA20ox1* in GA biosynthesis. Taken together, our findings demonstrate that *AtHSPR* plays a positive role in GA- and light intensity-mediated regulation of flowering and seed set.

## Teaser

*AtHSPR* acts as a positive regulator of flowering time and seed set partly through affecting the levels of gibberellin and regulating a light intensity-dependent pathway in Arabidopsis.

## Introduction

Different plants have evolved diverse flowering times to adapt to ecological niches. The change from vegetative to reproductive growth is sophisticatedly controlled by both endogenous developmental cues and external environmental stimuli to maximize reproductive success and seed production. The major pathways regulating flowering time have been widely reported in the model plant *Arabidopsis thaliana*. The photoperiod, vernalization, and ambient temperature pathways coordinate to regulate flowering through sensing environmental signals such as changes in day length and temperature. Other pathways fine-tune the flowering time, including the autonomous, gibberellin (GA), and age-associated pathways, which are independent of environmental cues (Fornara *et al.*, 2010; Blümel *et al.*, 2015; Wang *et al.*, 2017). In addition, a small number of floral integrator genes, including *SUPPRESSOR OF OVEREXPRESSION OF CONSTANS 1* (*SOC1*), *FLOWERING LOCUS T* (*FT*), *TWIN SISTER OF FT* (*TSF*), and *AGAMOUS-LIKE 24* (*AGL24*), activate the floral meristem identity genes *APETALA1* (*AP1*), *LEAFY* (*LFY*), *SEPALLATA 3* (*SEP3*), and *FRUITFULL* (*FUL*), which promote floral development processes (Zeevaart, 2008; Fornara *et al.*, 2010; Blümel *et al.*, 2015; Sharma *et al.*, 2016).

The phytohormone GA regulates plant growth and plays a predominant role in the promotion of flowering in Arabidopsis. The GA biosynthesis enzymes GA 20 OXIDASE (GA20ox) and GA 3 OXIDASE (GA3ox) catalyse multiple oxidation reactions to synthesize various GA intermediates and bioactive GAs, including GA_1_ and GA_4_ (Hu *et al.*, 2008; Sun, 2008; Yamaguchi, 2008). Conversely, active GAs can be deactivated by catabolic enzymes such as GA 2 OXIDASE (GA2ox). Mutations that either reduce the GA biosynthetic pathway or increase deactivation of GA result in a plant with a delayed flowering phenotype (Hu *et al.*, 2008; Sun, 2008; Song *et al.*, 2012; Sharma *et al.*, 2016). Under short-day (SD) conditions, GA acts as a positive regulator that directly promotes the expression of *SOC1* (Moon *et al.*, 2003), which subsequently activates the downstream genes *LFY* and *AP1* to promote flowering. The DELLA proteins REPRESSOR OF GA1-3 (RGA), REPRESSOR OF GA1-3-LIKE 1 (RGL1), and RGL2 repress GA signaling to delay floral development (Cheng *et al.*, 2004; Yu *et al.*, 2004). Mutation of one of the three GA receptors (GIBBERELLIC ACID-INSENSITIVE DWARF 1, GID1a, b, and c) decreases the expression levels of the floral integrator genes *FT* and *TSF*, resulting in a failure to flower under long-day (LD) conditions (Galvão *et al.*, 2012). The transcription factors SQUAMOSA PROMOTER BINDING PROTEIN-LIKE 3 (SPL3), SPL4, and SPL5 are direct targets of *SOC1* and *FT* (Jung *et al.*, 2012). Furthermore, SPL3 directly activates *AP1*, *LFY*, and *FUL* to promote floral transition, and its overexpression accelerates flowering (Yamaguchi *et al.*, 2009; Porri *et al.*, 2012). *SHORT VEGETATIVE PHASE* (*SVP*) encodes a MADS box transcription factor that not only reduces GA biosynthesis by repressing *GA20ox2* expression levels but also represses the expression of floral integrator genes in the vegetative meristem (Andrés *et al.*, 2014). The transcriptional repressor OVATE FAMILY PROTEIN1 (OFP1) represses cell elongation by controlling the expression of *GA20ox1* in Arabidopsis (Wang *et al.*, 2007). OFP1 has shown to be involved in regulating flowering time by interacting with a transcriptional activator BLH3 (BEL1-LIKE HOMEODOMAIN 3) or a BEL1-like homeodomain transcriptional repressor ATH1 (ARABIDOPSIS THALIANA HOMEOBOX1) (Wang *et al.*, 2016; Zhang et al., 2016, 2018). The transcription factors KNOX (Class-I KNOTTED1-LIKE HOMEOBOX) have been shown to reduce GA levels by promoting the expression the catabolic genes *GA2ox2* and *GA2ox4* or by directly repressing the transcription of the biosynthetic gene *GA20ox1* in the shoot apical meristem (SAM; Sakamoto *et al.*, 2001; Chen *et al.*, 2004; Jasinski *et al.*, 2005). The GA pathway also interacts with the photoperiodic pathway to regulate the expression of *FT* (Hisamatsu and King, 2008; Yamaguchi, 2008; Osnato *et al.*, 2012; Song *et al.*, 2012).

Flowering time is also influenced by light quality, including the ratio of red to far-red light (R:FR), the presence of blue light, and the light intensity. The red-light receptors PHYTOCHROME A (PHY-A) and PHY-B and the blue-light receptors CRYPTOCHROME 2 (CRY 2) and FLAVIN-BINDING, KELCH REPEAT, F-BOX 1 (FKF1) regulate the transcription of CONSTANS (CO) and PHYTOCHROME INTERACTING FACTORS (PIFs), which in turn regulate the expression of the floral inducer FT (Kobayashi and Weigel, 2007; Leivar and Quail, 2011; Andrés and Coupland, 2012). Compared to the amount of research on both light quality and photoperiod in relation to flowering time, there are fewer studies regarding the effects of light intensity. Arabidopsis plants grown under low-light intensity with a normal R:FR ratio show a delayed flowering phenotype, which is hypothesized to be partly due to the attenuation of photosynthesis and the inhibition of photosynthate transport under low light (Thomas, 2006; King *et al.*, 2008). Under low light, *MYB-RELATED PROTEIN 1* (*MYR1*) and *MYR2* act as redundant repressors of flowering and negatively regulate the levels of bioactive GAs (Zhao *et al.*, 2011).

*AtHSPR* encodes a nuclear-localized protein with ATPase activity, named *SMXL4* in the Col-0 ecotype, and is a member of the *SMXL* family of eight genes in Arabidopsis consisting of *SUPPRESSOR OF MORE AXILLARY GROWTH2* (*SMAX*) and *SMAX-LIKE* (*SMXL*) genes (Khosla and Nelson, 2016). SMAX1 and SMXL2 have been reported to mediate strigolactone (SL) and karrikin (KAR) responses to regulate seed germination and seedling development (Stanga et al., 2013, 2016). The functions of SL- and KAR-independent SMXL3, 4, and 5 are essential for the regulation of phloem formation (Wallner *et al.*, 2017). SMAX1 and SMXL6, 7, and 8 have been demonstrated to regulate shoot development by repressing SL and KAR signaling (Soundappan *et al.*, 2015; Wang *et al.*, 2015; Liang *et al.*, 2016). In our previous work we showed that *AtHSPR* is involved in the ABA-mediated stress response in Arabidopsis (Yang *et al.*, 2015), but the mechanisms by which it controls plant growth and development are not well understood. In this study, we further characterized an *Athspr* mutant and the biological functions of *AtHSPR* in flowering and seed set. The *Athspr* mutant exhibited delayed flowering and reduced fertility, which were strongly consistent with altered GA biosynthesis and signaling. In addition, we found that *AtHSPR* plays a role in light intensity-dependent regulation of flowering time, reproductive organ development, and seed production. The AtHSPR protein may form a complex with the OFP1 and KNAT5, which function as repressors of the transcription of *GA20ox1* in GA biosynthesis. Our results indicate that *AtHSPR* is responsible for GA- and light intensity-mediated control of flowering time and seed set.

## Materials and methods

### Plant materials

The *Athspr* mutant and transgenic plants used in this study were derived from the *Arabidopsis thaliana* C24 ecotype. The mutant *Athspr*, *Pro-AtHSPR::AtHSPR*, and *35S::AtHSPR* lines have been described previously by Yang *et al.* (2015). A 5700-bp genomic sequence containing both the promoter (2431 bp, predicted by the web-database PLACE; Higo *et al.*, 1999) and the entire *AtHSPR* gene (3269 bp) was transformed into the *Athspr* background to generate an *AtHSPR* complementation line (COM). All homozygous T_3_ transgenic plants were used for further analyses. Seeds were surface-sterilized with 70% (v/v) ethanol for 50 s and then with 1% NaClO for 5 min, washed extensively four times with sterilized water, and sown on Murashige and Skoog (MS) medium (pH ~5.8–6.0) with 0.8% (w/v) agar and 1% sucrose. After vernalization for ~48–72 h in the dark at 4 °C, plants were grown at ~21–22 °C with ~60–70% relative humidity under either long-day conditions (LD; 16/8-h light/dark, with different light intensities between 20–150 µmol m^–2^ s^–1^) or short-day conditions (SD; 8/16-h light/dark, at 150 µmol m^–2^ s^–1^). At 7 d old, seedlings were transferred to soil and continued to be grown under the same conditions.

### Phenotypic analyses

Flowering time was determined by counting the days after sowing at the time of bolting and the total number of leaves (rosette and cauline leaves) formed before the first flower. Three independent experiments for performed and a total of at least 40 individual soil-grown plants were used.

The leaf angle between the horizontal plane and the petiole of the most erect, fully expanded leaf of each plant was measured under SD and LD conditions. The hypocotyl length of 7-d-old seedlings was measured from the base of the cotyledons to the top of the first lateral root under various light conditions (red, blue, far-red, low-light/LD conditions, SD, and dark) (Yuan *et al.*, 2007). All measurements were repeated three times, and at least 25 seedlings were measured each time for each genotype. The siliques were divided into four types according to the rate of seed set, and the percentage of each type on the primary inflorescence stem was determined.

Transgenic plants carrying the *Pro-AtHSPR::GUS* reporter construct in the C24 background were used for expression analysis as described previously (Yang *et al.*, 2015).

### Microscopic imaging

The floral transition in the SAM was observed using SEM. Fresh tissue samples were dissected under a microscope and fixed to specimen stubs using a carbon adhesive, before being frozen in liquid nitrogen for ~1–2 min. The frozen samples were then immediately observed using a TM-3000 tabletop SEM (Hitachi). The development of the SAM was monitored according to how many leaves the plant had produced (Tal *et al.*, 2017), while the floral stages were monitored according to Alvarez-Buylla *et al.* (2010).

The SAMs and the siliques of the wild-type (WT) and *Athspr* mutant were fixed in FAA (formaldehyde, acetic acid, alcohol) at 4 °C for 24 h, rehydrated in an ethanol series, and stained in 0.1% safranin for 40–48 h at 25 °C. The samples were then dehydrated in a gradient of ethanol, embedded in paraffin, cut longitudinally using a microtome, dewaxed in xylene, rehydrated, counterstained with 0.1% Brilliant Green, and dehydrated again. The samples were then imaged using a digital microscope (Leica Microsystems). The SAM base was defined as the location of the leaf primordium, and the height of the SAM was measured as the distance from the top of the SAM to the base using the ImageJ software (https://imagej.nih.gov/ij/).

### RNA extraction and quantitative real-time PCR

Total RNA was isolated using a MiniBEST Plant RNA Extraction Kit (Takara) and subjected to reverse-transcription using PrimeScript^TM^RT Master Mix (Takara) following the manufacturer’s instructions. Quantitative real-time PCR (qRT-PCR) analyses were performed using a SYBR Premix ExTaq II kit (Takara) using a Bio-Rad CFX96 real-time system with gene-specific primers (Supplementary Table S1 at *JXB* online). *PROTEIN PHOSPHATASE 2A* (*PP2A*) or *UBQ10* was used as the internal control. All experiments were performed with three biological replicates and three technical replicates. The relative expression of the target genes was calculated according to the 2^–∆∆*C*T^ method (Schmittgen and Livak, 2008).

### Exogenous GA_3_ application assays

A stock solution of GA_3_ (Sigma) was prepared in 100% ethanol, from which a treatment solution of 100 µM GA_3_ plus 0.02% Silwet-77 in water was made. Control plants received a mock treatment of 0.02% Silwet-77 in water only. For plants grown under LD conditions treatment started when plants were 21 d old and for plants grown under SD conditions treatment started when plants were 32 d old. Treatment consisted of spraying twice a week for 3 weeks until the onset of flowering.

### Measurement of GA pathway intermediates

Plant material (~150 mg FW) was frozen in liquid nitrogen, ground into powder, and extracted with 1 ml of 80% methanol at 4 °C for 12 h. The extract was centrifuged at 12 000 *g* at 4 °C for 15 min. The supernatant was collected, evaporated to dryness under a nitrogen gas stream, and reconstituted in 100 ml of 95% acetonitrile. The solution was centrifuged again at 12 000 *g* at 4 °C for 15 min, and the supernatant was collected for LC-MS analysis. The sample extracts were analysed using an LC/MS/MS system (HPLC, Shim-pack UFLC SHIMADZU CBM30A system; MS, Applied Biosystems 4500 Q TRAP) controlled by Analyst 1.6 software (AB Sciex). The HPLC was carried out on a Waters ACQUITY UPLC HSS T3 C18 column (1.8 µm I.D., 2.1 × 100 mm). Solvent A was water with 0.04% acetic acid and solvent B was acetonitrile with 0.04% acetic acid. The gradient program was 100% A at 0 min, ramped to 5% A by 11.0 min, back to 95% A by 12.1 min, and held until 15.0 min. The flow rate was 0.4 ml min^–1^. The temperature was set at 40 °C. The effluent was alternatively connected to an ESI-triple quadrupole-linear ion trap (Q TRAP)-MS. Several GAs and GA intermediates were further analysed, namely GA_1_, GA_4_, GA_7_, GA_9_, GA_15_, GA_19_, GA_20_, GA_24_, and GA_53_.

### Measurement of pistil and filament lengths

Flowers of the WT, *Athspr*, *Pro-AtHSPR::AtHSPR*, and *35S::AtHSPR* lines grown under LDs with a range of light intensities (20–150 µmol m^–2^ s^–1^) were imaged under a microscope (Leica Microsystems) at floral stage 13. The lengths of the pistil and of the longest of the four filaments for each flower were measured using the ImageJ software.

### Alexander staining

Alexander staining was used to distinguish normal pollen grains from aborted ones according to the intensity of color. Flower buds at stage 12 were collected from the WT, *Athspr*, *Pro-AtHSPR::AtHSPR*, and *35S::AtHSPR* lines and fixed in 1 ml of Carnoy’s solution (6:3:1 v/v ethanol:chloroform:acetic acid) for at least 2 h. The fixative was removed before the buds were dissected to obtain individual anthers. These were then submerged completely with an appropriate volume of Alexander staining solution and incubated at 25 °C for 2 h. The stained anthers were visualized and imaged using a digital stereo microscope (SteREO Discovery V20, Zeiss).

### Transcriptional activity assays

Transient GUS expression was employed to examine the transcriptional activity of AtHSPR. *AtHSPR* fused with *GAL4DB* under the control of the *CaMV35S* promoter was used as an effector (*35S::GAL4DB-AtHSPR*) (Hellens *et al.*, 2005). The GUS gene, driven by four copies of upstream GAL4 DNA binding sites [*GAL4(4x)-D1-3(4x)*], was used as a reporter (*GAL4(4x)-D1-3(4x)-GUS*) (Tiwari *et al.*, 2001), and *LUC* under the control of the 35S promoter was used as the internal control (*35S::LUC*). The reporter and effector plasmids were co-transformed into Arabidopsis protoplasts using a PEG-mediated transformation method previously described by Yoo *et al.* (2007). GUS activity was measured using a Plant GUS ELISA Kit (Laibio, China), and LUC activity was measured using an Amplite™ Luciferase Reporter Gene Assay Kit (AAT Bioquest, USA). Data are presented as GUS/LUC ratios. the primers used for plasmid construction are shown in Supplementary Table S1.

### Yeast two-hybrid assays

The Matchmaker Gold Yeast Two-Hybrid System (Clontech) was used to screen candidate proteins that interact with AtHSPR using yeast mating. Full-length coding sequences of *AtHSPR*, *KNAT5*, and *OFP1* were cloned into the vector pGBKT7 (BD-AtHSPR, BD-KNAT5, and BD-OFP1) or the vector pGADT7 (AD-KNAT5 and AD-OFP1), and co-transformed into the Y2HGold yeast strain using the lithium acetate-mediated method (Yeastmaker™ Yeast Transformation System 2, Clontech). The transformed yeast cells were plated on appropriate double-dropout (DDO) medium without leucine and tryptophan (SD/–Leu/–Trp) at 30 °C for 3 d. The interactions in yeast were tested on quadruple-dropout (QDO) medium without Leu, Trp, adenine, and histidine (SD/–Ade/–His/–Leu/–Trp) and quadruple-dropout medium supplemented with aureobasidin A (AbA) and X-α-gal (QDO/AbA/X-α-gal). The growth was recorded after incubation for 4 d at 30 °C. The plasmids AD (activation domain) and BD (binding domain) were transformed into Gold yeast cells and used as a negative control. The primers used for plasmid construction are shown in Supplementary Table S1.

### GST pull-down assays

The full-length coding sequences of *AtHSPR*, *KNAT5*, and *OFP1* were fused with either an N-terminal glutathione *S*-transferase (GST) tag using a pGEX-6P-1 vector or a C-terminal His tag using a pET-30a vector to express GST-AtHSPR, GST-KNAT5, His-KANT5, and His-OFP1. The fused constructs were transformed into the *Escherichia coli* Rosetta strain for protein expression. The GST-tagged recombinant proteins were purified with glutathione (GSH) magnetic beads (70601, Beaver-Bio) according to the manufacturer’s instructions. The primers used for plasmid construction are shown in Supplementary Table S1.

For GST pull-down assays, the GSH magnetic beads bound with GST or GST-proteins were rinsed five times with washing buffer (140 mM NaCl, 2.7 mM KCl, 10 mM Na_2_HPO_4_, 1.8 mM KH_2_PO_4_, pH 7.4, 2 mM dithiothreitol, 1.5% Triton X-100, and 1 mM PMSF) and then the His-proteins were incubated with pre-washed GST-bound or GST-protein-bound GSH magnetic beads in lysis buffer (140 mM NaCl, 2.7 mM KCl, 10 mM Na_2_HPO_4_, 1.8 mM KH_2_PO_4_, pH 7.4, and 1 mM PMSF) for 2 h at 4 °C with gentle shaking. After incubation, the magnetic beads were rinsed five times with the washing buffer. The magnetic beads were then boiled with 2×SDS loading buffer for 10 min, and then the pulled-down proteins were subjected to immunoblotting analysis with anti-His and anti-GST antibodies (Abmart, M20001 and M20007, respectively).

### Statistical analysis

All data are presented as means (±SD) of three independent experiments (*n*=3). Significant differences between pairs of means were determined using Student’s *t*-test. For multiple comparisons, statistical significance was determined using ANOVA followed by Duncan’s Multiple Range Test using SPSS Statistics (IBM, USA).

## Results

### AtHSPR positively regulates flowering time under both long- and short-day conditions

**Fig. 1.**
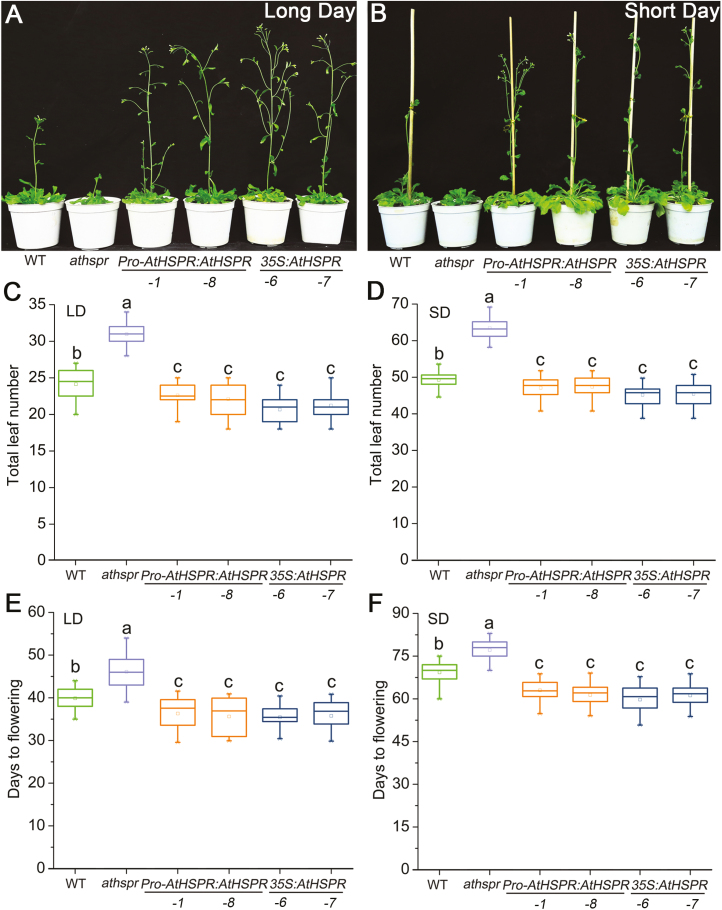
AtHSPR positively regulates ﬂowering under long- and short-day conditions. (A, B) Flowering phenotypes of 42-day-old plants of the Arabidopsis wild-type (WT), *Athspr* mutant, *Pro-AtHSPR::AtHSPR-1* and *-8*, and *35S::AtHSPR*-*6* and *-7* lines grown under long-days (LDs; 16/8 h photoperiod) and (B) 70-d-old plants grown under short days (SDs; 8/16 h photoperiod). The light intensity was 150 µmol m^–2^ s^–1^. The images are representative of at least three separate experiments. (C, D) Total leaf number (rosette and cauline leaves) of the plants grown under either (C) LDs or (D) SDs. (E, F) Number of days to flowering of the plants grown under either (E) LDs or (F) SDs The data are shown as box plots with upper and lower quartiles and the median; *n*=40. Different letters indicate significant differences as determined using ANOVA followed by Duncan’s multiple range test (*P*<0.05).

The *Athspr* mutant has previously been determined to contain a T-DNA insertion at the end of the third exon of *AtHSPR*, which disrupts production of a full-length transcript (Yang *et al.*, 2015). Compared with the WT, the *Athspr* mutant had darker green leaves, a shorter stature, a later flowering time, shorter siliques, and reduced seed set (Fig. 1A, B). Complementation of the mutant with the *Pro-AtHSPR::AtHSPR* construct rescued these defective phenotypes (Supplementary Fig. S1). To explore the functions of *AtHSPR*, transgenic *Pro-AtHSPR::AtHSPR* and *35S::AtHSPR* plants were generated in the wild-type C24 background. The flowering times of the WT, *Athspr*, *Pro-AtHSPR::AtHSPR*, and *35S::AtHSPR* plants were determined by counting both the total number of leaves formed before the first flower appeared and the number of days to flowering under LD and SD conditions. Compared with the WT, flowering in the *Athspr* mutant was significantly delayed under both LDs and SDs (Fig. 1). In contrast, the *Pro-AtHSPR::AtHSPR-1* and *-8* lines and the *35S::AtHSPR-6* and *-7* lines ﬂowered earlier than WT plants under LDs and SDs. The differences were greater under SDs (Fig. 1B, D, F). These results suggested that AtHSPR positively regulated flowering time under LD and SD conditions. Transgene abundance was examined using qRT-PCR (Supplementary Fig. S2). There were differences in *AtHSPR* expression levels between the *Pro-AtHSPR::AtHSPR* and *35S::AtHSPR* lines, even though no significant differences had been observed in flowering time. Since the phenotype of *Pro-AtHSPR::AtHSPR-8* was similar to the other *Pro-AtHSPR::AtHSPR* and *35S::AtHSPR* lines, and it showed a moderate level of over-expression of *AtHSPR*, *Pro-AtHSPR:AtHSPR-8* was mostly used as the experimental material for further analysis.

Enlargement and doming of the SAM are hallmarks of the transition from the vegetative to reproductive phase (Tal *et al.*, 2017). The morphology of the SAM in WT, *Athspr*, and *Pro-AtHSPR:AtHSPR-8* plants at different developmental stages were observed using SEM (Supplementary Fig. S3A). After producing 12 leaves (12L), the SAMs of both the WT and *Athspr* were flat, whereas that of *Pro-AtHSPR::AtHSPR-8* exhibited a fully triangular dome-shaped cone. At 14L, the SAM in the WT also exhibited a fully triangular dome-shaped cone, while that of *Athspr* was still flat. By 16L, the SAM in the WT was domed, the SAM in *Pro-AtHSPR::AtHSPR-8* was fully developed, but the SAM in *Athspr* was only slightly domed. These observations were consistent with those of paraffin sections of the WT and *Athspr* plants (Supplementary Fig. S3B). The *Athspr* mutant plants had a smaller SAM before the transition from the vegetative to reproductive stage compared with the WT; however, the height and width of the SAM in the *Athspr* mutant when it had produced 16 leaves were not significantly different from the WT when it had produced 14 leaves, which is the transition from vegetative to reproductive stage. This implied that the delayed enlargement and doming of the SAM in the mutant may have been associated with a delay in floral transition (Supplementary Fig. S3B–D). The SEM observations also showed that the pistil at floral stage 12 in the mutant was thicker and shorter whilst the stigma was slightly shorter (Supplementary Fig. S4B, inset) compared with the WT (Supplementary Fig. S4A–C). The gynoecium of the WT consisted of two fused carpels, and the mutant showed no obvious differences in carpel development in either the SEM or paraffin sections, except that the siliques of *Athspr* were stubby under LDs (Supplementary Fig. S1C, D, S4).

### *AtHSPR* is expressed in a tissue-specific manner

We have previously reported that the *AtHSPR* promoter is highly active in the vascular tissues of all organs (Zhang *et al.*, 2014). To further examine its tissue-specific expression during plant development, histochemical GUS assays were performed using *Pro-AtHSPR::GUS* plants. GUS activity was mainly observed in the vasculature of hypocotyls, cotyledons, mature leaves, and the shoot apex (Supplementary Fig. S5A–E). GUS staining was also observed in the stamens and pistils of flowers, especially in the vascular tissues of the sepals, petals, filaments, the style of the flowers, and the replum, valve, and septum of siliques in mature plants (Supplementary Fig. S5F–M). These results indicated that *AtHSPR* was expressed in the vascular tissue and floral organs in a tissue-specific manner.

### Exogenous application of GA_3_ partially rescues late flowering and low seed set in the *Athspr* mutant under both LD and SD conditions

**Fig. 2.**
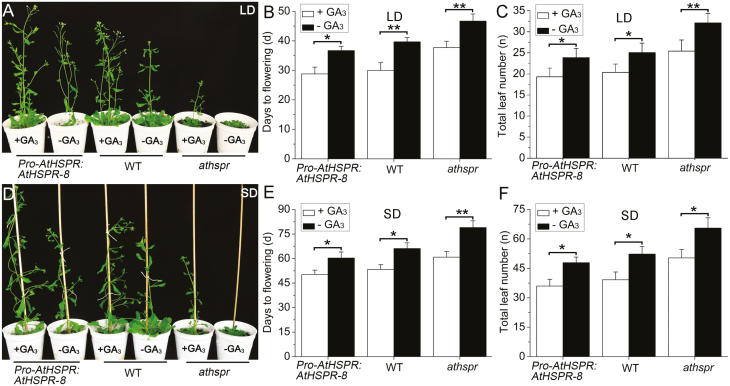
Exogenous application of GA_3_ partially rescues the late-flowering phenotype of the *Athspr* mutant grown under both long-day and short-day conditions. Plants of the Arabidopsis wild-type (WT), *Athspr* mutant, and *Pro-AtHSPR::AtHSPR-8* line were grown under either long-days (LDs; 16/8 h photoperiod) or short days (SDs; 8/16 h photoperiod) at a light intensity of 150 µmol m^–2^ s^–1^ and sprayed twice a week for 3 weeks with a solution either with or without 100 µM GA_3_. Spraying began when the plants were either 21 d old for LDs or 32 d old for SDs. (A, D) Phenotypes of the plants at (A) 42 d old for the LD treatment and (D) 53 d old for the SD treatment. (B, E) Days to flowering for (B) the LD treatment and (E) the SD treatment. (C, F) Total leaf number (rosette and cauline leaves) at flowering for (C) the LD treatment and (F) the SD treatment. Data are means (±SD) of *n*=30 replicates. Significant differences between means were determined using Student’s *t*-test: *P<0.05, **P<0.01.

GA plays an important role in floral induction in Arabidopsis, and we found mutation of *AtHSPR* resulted in phenotypes resembling those associated with partial GA deficiency, including semi-dwarfism, dark-green leaves, and male sterility (Koornneef and van der Veen, 1980; Hu *et al.*, 2008; Plackett *et al.*, 2011). To examine whether the late-flowering phenotype of the *Athspr* mutant was related to alterations in GA biosynthesis and/or signal transduction, we examined the responses of WT, *Athspr*, and *Pro-AtHSPR::AtHSPR-8* plants to exogenously applied GA_3_. Under both LDs and SDs, exogenous GA_3_ could partially rescue the *Athspr* phenotypes, shortening its time to flowering (Fig. 2). *Pro-AtHSPR::AtHSPR-8* plants showed similar responses to exogenous GA application as the WT under both LDs and SDs. These results suggested that the late-flowering phenotype of *Athspr* may be partially due to a perturbation of endogenous GAs.

**Fig. 3.**
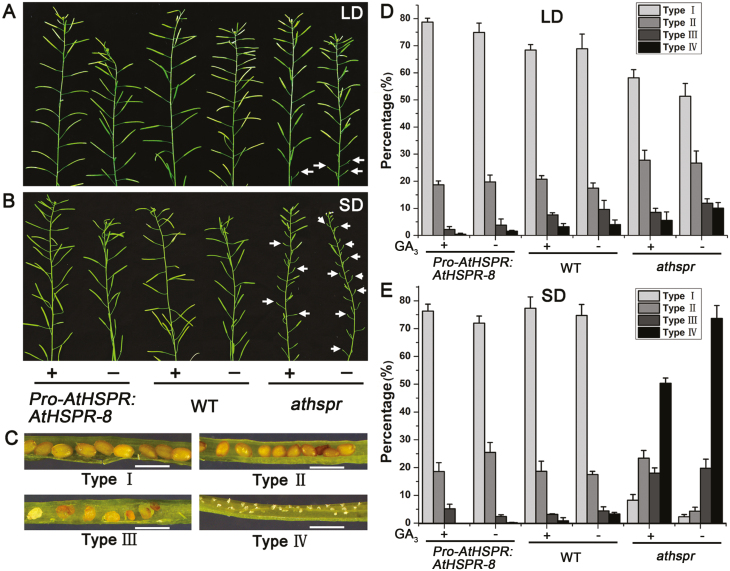
*AtHSPR* influences GA-regulated silique and seed development. Plants of the Arabidopsis wild-type (WT), *Athspr* mutant, and *Pro-AtHSPR::AtHSPR-8* line were grown under either long-days (LDs; 16/8 h photoperiod) or short days (SDs; 8/16 h photoperiod) at a light intensity of 150 µmol m^–2^ s^–1^ and sprayed twice a week for 3 weeks with a solution either with or without 100 µM GA_3_. Spraying began when the plants were 21 d old under LDs and 32 d old under SDs. (A, B) Primary stems bearing siliques of on plants with (+) or without (–) GA_3_ treatment under (A) LDs and (B) SDs. The arrows indicate empty siliques in the *Athspr* mutant. (C) Siliques were classified into four categories: Type I, siliques were long and seeds were filled; Type II, siliques were a little shorter than Type I and most seeds were filled; Type III, seed number per silique was significantly decreased and most seeds were aborted; Type IV, siliques were remarkably short and all seeds were undeveloped. Scale bars are 1 mm. (D, E) The percentages of each type of silique in the different lines with (+) or without (–) GA_3_ treatment under (D) LDs and (E) SDs. Data are means (±SD) of *n*=10 replicates.

GA is also essential for the development of siliques (Hu *et al.*, 2008; Sun, 2010). To investigate the effects of *AtHSPR* on GA-mediated development of siliques, we examined seed set after treatment with GA_3_ under both LD and SD conditions. Compared with the WT, the *Athspr* mutant had a greater number of siliques with dramatically reduced length and lower seed set under both LD and SD conditions (Fig. 3A, B). We classified the siliques into four categories (Fig. 3C), as follows. Type I, siliques were long and seeds were filled; Type II, siliques were a little shorter than Type I and most seeds were filled; Type III, seed number per silique was significantly decreased, and most seeds were aborted; and Type IV, siliques were remarkably short and all seeds were undeveloped, leaving empty spaces in the silique. Types I and II were considered to be normal in terms of seed set. In *Athspr*, the rate of aborted and undeveloped seeds in Type III and IV siliques was 22% under LDs and 93.3% under SDs, with nearly 73.6% of the siliques being completely empty of developing seeds in the latter (Fig. 3E). On the primary stem of the mutant, the uppermost 15 siliques were mostly sterile, with only one or two carrying any filled seeds under SDs. This suggested that the mutation of *AtHSPR* had significant effects on fertility under SDs. In contrast, the rate of abnormal siliques (Types III and IV) was lower in the *Pro-AtHSPR::AtHSPR-8* plants (2.7%) compared to WT (7.8%) under SDs. Exogenous application of GA_3_ partially restored silique development and seed set in the *Athspr* mutant (Fig. 3). The number of seed-filled siliques (Types I and II) in *Athspr* increased by about 20% after GA treatment under SDs (Fig. 3E), and the seed set in the uppermost siliques was improved (Fig. 3B). These results suggested that *AtHSPR* was at least partially involved in GA-mediated development of siliques.

### GA biosynthesis and metabolism are altered in the *Athspr* mutant

**Fig. 4.**
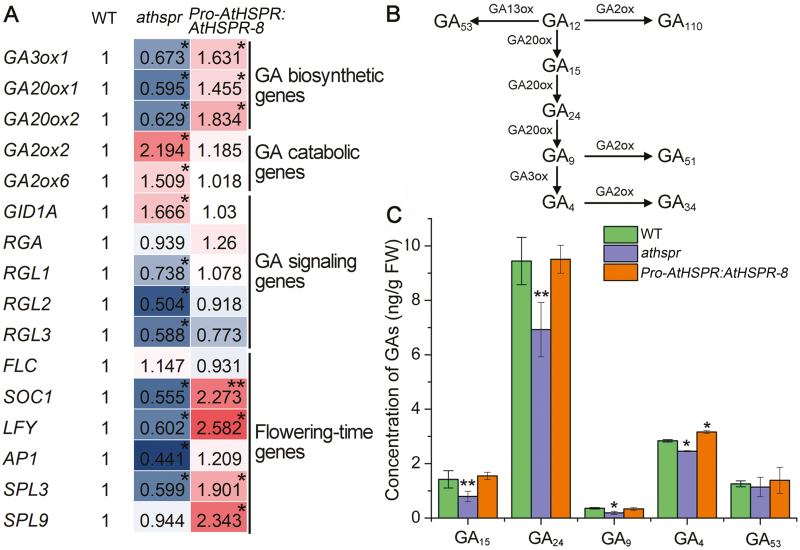
GA biosynthesis in apical tissues of the Arabidopsis wild-type (WT), *Athspr* mutant, and *Pro-AtHSPR::AtHSPR-8* line at the mature stage. Measurements were made on 8-week-old plants grown under short-day conditions (8/16 h light/dark) at a light intensity of 150 µmol m^–2^ s^–1^ (A) qRT-PCR analyses of GA biosynthetic and catabolic genes, GA signaling genes, and flowering-time genes. Total RNA was isolated from the meristem tissues. Expression levels are relative to those of the WT, which were set as 1. Data are means of *n*=3 replicates. (B) Schematic representation of the non-13-hydroxylated GA biosynthetic pathway in Arabidopsis. (C) Endogenous concentration of GAs in the plants. Apical tissues including the meristem and a few small leaves were harvested. Data are means (±SD) of *n*=3 replicates. In (A, C) significant differences compared to the WT were determined using Student’s *t*-test: *P<0.05, **P<0.01.

GA plays a crucial role in promoting flowering, particularly under SDs (Wilson *et al.*, 1992). We therefore compared the expression levels of several key genes that contribute to GA biosynthesis, catabolism, and signaling in the WT, *Athspr*, and *Pro-AtHSPR::AtHSPR-8*. Total RNA was isolated from the apical tissues of 8-week-old plants grown under SDs, including the meristem and a few small leaves, and subjected to qRT-PCR analysis (Fig. 4A). Compared to the WT, genes involved in GA biosynthesis (*GA20ox1*, *GA20ox2*, and *GA3ox1*) were down-regulated in the *Athspr* mutant, whereas those contributing to GA catabolism (*GA2ox2* and *GA2ox6*) and signaling were up-regulated. In *Pro-AtHSPR::AtHSPR-8* plants, the expression levels of GA biosynthesis-related genes were up-regulated and those of catabolism-related genes were down-regulated. In *Athspr* plants, the expression levels of GA signaling genes (*RGL1–3*) were decreased, whilst the expression of a GA receptor gene (*GID1A*) was up-regulated compared to the WT, suggesting feedback regulation of these genes. The expression levels of genes related to flowering time such as *SOC1*, *LFY*, *AP1*, and *SPL3* (all of which contribute to promotion of flowering) were lower in *Athspr* than in the WT under SDs. In contrast, *Pro-AtHSPR::AtHSPR-8* plants showed relatively higher expression of these genes compared with the WT.

The qRT-PCR results showed that the expression levels of GA biosynthesis genes were significantly lower in the *Athspr* mutant than in the WT whilst the expression levels of GA catabolism genes were higher. Preliminary testing showed that the mutant also contained lower levels of GAs, possibly due to a reduction of GA biosynthetic activity in the early steps of the pathway or to increased activity in an early catabolic step (Fig. 4B). The concentrations of the main GA biosynthetic and catabolic intermediates were quantified from apical tissues of 8-week-old WT, *Athspr*, and *Pro-AtHSPR::AtHSPR-8* plants (Fig. 4C). Although reliable quantification of GA_1_, GA_7_, GA_19_, and GA_20_ was confounded by extremely low levels of these metabolites, we were able to reliably measure GA_4_, three of its precursors (GA_15_, GA_24_, and GA_9_), and one product of GA_12_ (GA_53_). Consistent with the late-flowering and decreased seed-set phenotypes of *Athspr*, the levels of GA_4_ and the intermediates that predominantly contribute to its biosynthesis (i.e. GA_15_, GA_24_, and GA_9_) were significantly reduced in the *Athspr* mutant compared with the WT under SDs. This may have been a direct consequence of the altered expression levels of *GA3ox1*, *GA20ox1*, and *GA20ox2* in *Athspr* (Fig. 4A, C). These results suggested that perturbation of the levels of GA biosynthetic and catabolic genes was a key consequence of the *AtHSPR* mutation, leading to decreased levels of GA and hence the late-flowering phenotype of *Athspr*.

### *AtHSPR* is involved in the control of light intensity-mediated flowering time

Light is of great importance in modulating flowering time (Zhou *et al.*, 2014). Given that the *Athspr* mutant had a prolonged vegetative phase under both LD and SD conditions, a severe late-flowering phenotype, and reduced fertility under SDs, we investigated whether the late-flowering phenotype was related to various responses to light signals in the plant. The expression of *AtHSPR* showed cyclical changes in response to photoperiod under both LDs and SDs (Supplementary Fig. S6). The *Athspr* mutant exhibited decreased leaf angle when compared to the WT under both LD and SD conditions (Supplementary Fig. S7). The responses of the WT, *Athspr*, and *Pro-AtHSPR::AtHSPR-8* were assessed under various light conditions, namely red, blue, far-red, low-light/LD, SD, and darkness (Supplementary Fig. S8). The hypocotyl length of *Athspr* and *Pro-AtHSPR::AtHSPR-8* seedlings were almost indistinguishable from the WT under continuous red, blue, and far-red light conditions, implying that *AtHSPR* does not participate in plant photomorphogenesis. However, the hypocotyl length of *Athspr* seedlings was shorter than the WT under SD, dark, and low-light/LD conditions, suggesting that the biological role of *AtHSPR* in regulating growth processes may be related to light intensity (Supplementary Fig. S9).

**Fig. 5.**
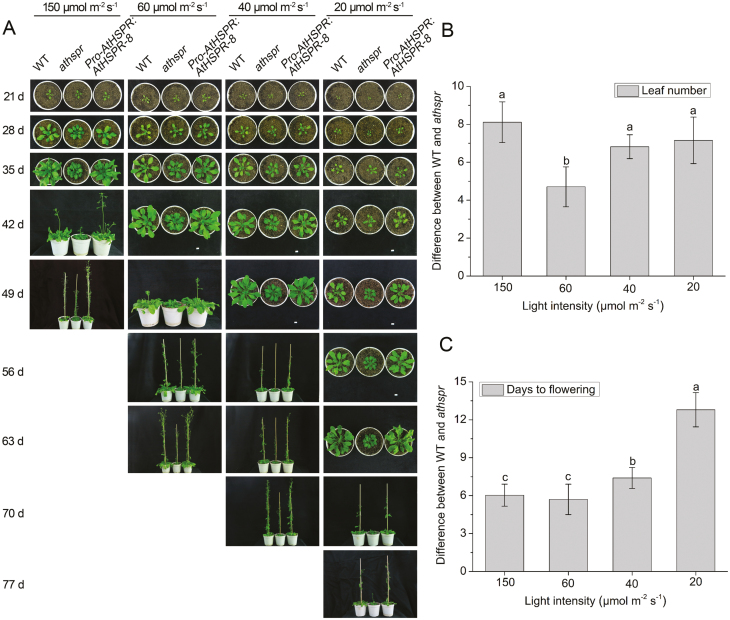
Differences in flowering time of the Arabidopsis wild-type (WT), *Athspr* mutant, and *Pro-AtHSPR::AtHSPR-8* plants grown under long-day conditions (16/8 h light/dark) with different light intensities. (A) Phenotypes of the plants at different growth stages and under different light intensities. (B) Difference in the mean number of leaves at the time of flowering between the WT and the *Athspr* mutant at the different light intensities, and (C) difference in the mean number of days to flowering at the different light intensities. Data are based on means (±SD) of *n*=3 independent experiments. Significant differences between means were determined using ANOVA followed by Duncan’s multiple range test (*P*<0.05).

**Fig. 6.**
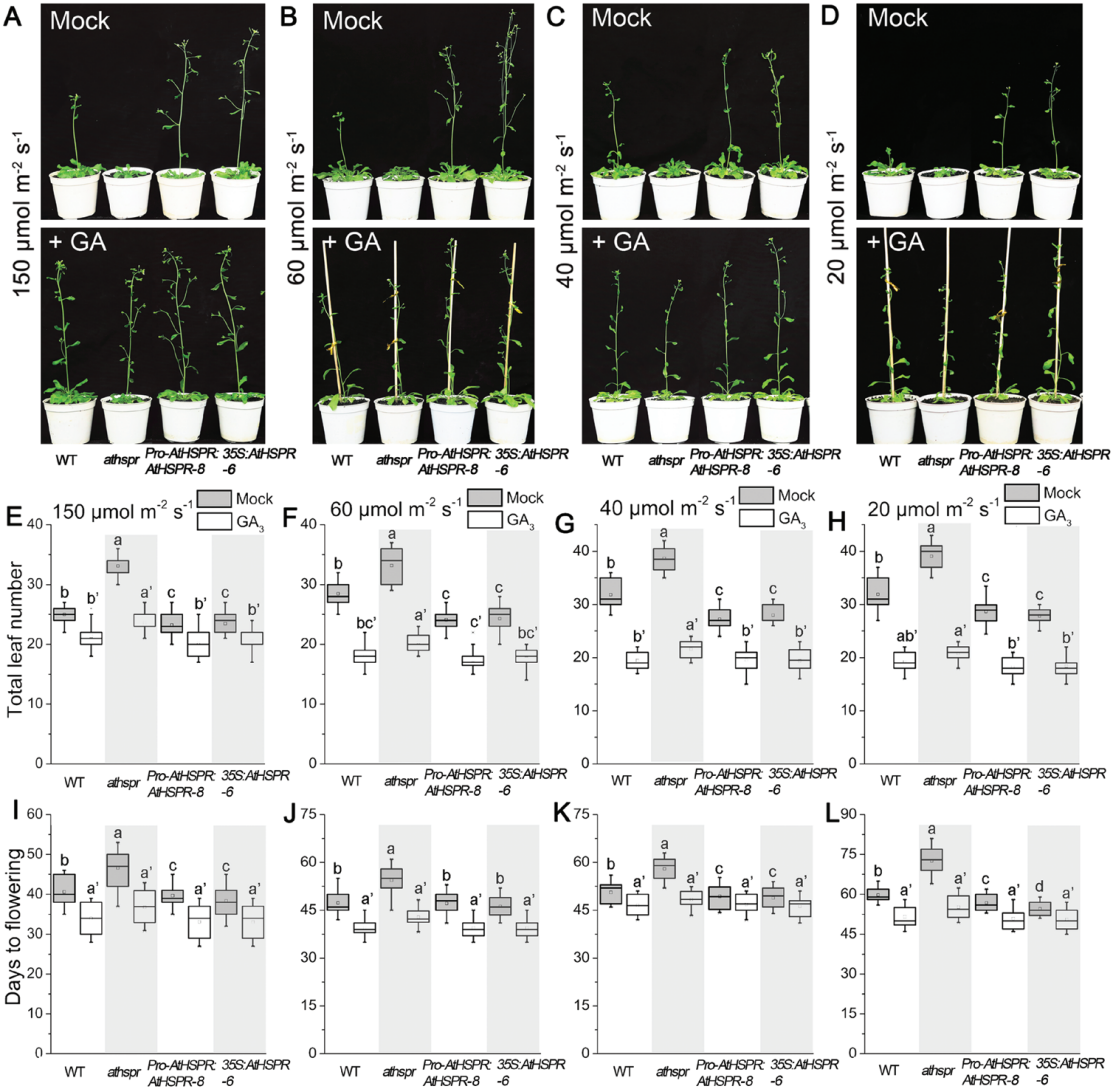
The Arabidopsis *Athspr* mutant displays defects in light intensity-dependent regulation of flowering time under long-day conditions (16/8 h light/dark). (A–D) The flowering phenotypes of the wild-type (WT), *Athspr*, *Pro-AtHSPR::AtHSPR-8*, and *35S::AtHSPR-6* plants grown under different light intensities and sprayed twice a week until the onset of flowering with a solution either with or without 100 µM GA_3_. Spraying began when the plants were 21 d old. The images were taken at (A) 40 d (Mock) and 39 d (+GA_3_), (B) 49 d (Mock) and 42 d (+GA_3_), (C) 55 d (Mock) and 49 d (+GA_3_), and (D) 65 d (Mock) and 60 d (+GA_3_). (E–H) Total leaf number at the time of flowering for plants grown under the different light intensities and with or without GA_3_ treatment. (I–L) Days to flowering for plants grown under the different light intensities and with or without GA_3_ treatment. The data are shown as box plots with upper and lower quartiles and the median; *n*=40. Different letters indicate significant differences as determined using ANOVA followed by Duncan’s multiple range test (*P*<0.05). Letters marked with a prime (´) refer to the Mock control.

The phenotypes of WT, *Athspr*, *Pro-AtHSPR::AtHSPR-8* and *35S::AtHSPR-6* plants were then observed under a range of light intensities. The phenotypes of the *Athspr* mutant were light-intensity dependent under LDs. Compared to the WT, the *Athspr* mutant reproducibly exhibited a late-flowering phenotype that showed increasing severity as the light intensity decreased (Figs 5, 6). Under high light intensity (150 µmol m^–2^ s^–1^), the *Athspr* plants flowered when they had a mean of 8.1 leaves more than when the WT flowered (Fig. 5B). Moderate light intensity (60 μmol m^-2^ s^-1^) decreased this difference to 4.7 leaves, but lower intensities had no significant effect compared with high light. In terms of days to flowering, there was a greater difference between the flowering times of the WT and *Athspr* with decreasing light intensity. Under high light, *Athspr* plants flowered 6.0 d later than the WT, and this increased to 12.8 d later under 20 μmol m^–2^ s^–1^ (Fig. 5C). In contrast, the flowering time in *Pro-AtHSPR::AtHSPR-8* plants was earlier than in the WT under 20 μmol m^-2^ s^-1^) (Figs 5A, 6D, H, L). Whilst these results were in agreement with previously reported negative effects of low light intensity on flowering (King *et al.*, 2008; Zhao *et al.*, 2011), they may have been due to the difference in the duration of leaf initiation (plastochron) between the WT and the *Athspr* mutant under these conditions (Wang *et al.*, 2008).

To investigate possible links between GA, *AtHSPR*, and light intensity in regulating flowering time, we examined whether the *Athspr*, *Pro-AtHSPR::AtHSPR-8*, and *35S::AtHSPR-6* lines had an altered response to GA under low-light (Fig. 6). *Pro-AtHSPR::AtHSPR-8* and *35S::AtHSPR-6* plants responded to GA_3_ application to a similar extent as the WT. Application of GA_3_ partially rescued the late-flowering in the *Athspr* mutant under low-light conditions, suggesting that this phenotype was at least in part related to defects in the GA pathway.

### Mutation of *AtHSPR* significantly affects seed fertility under low light intensity

**Fig. 7.**
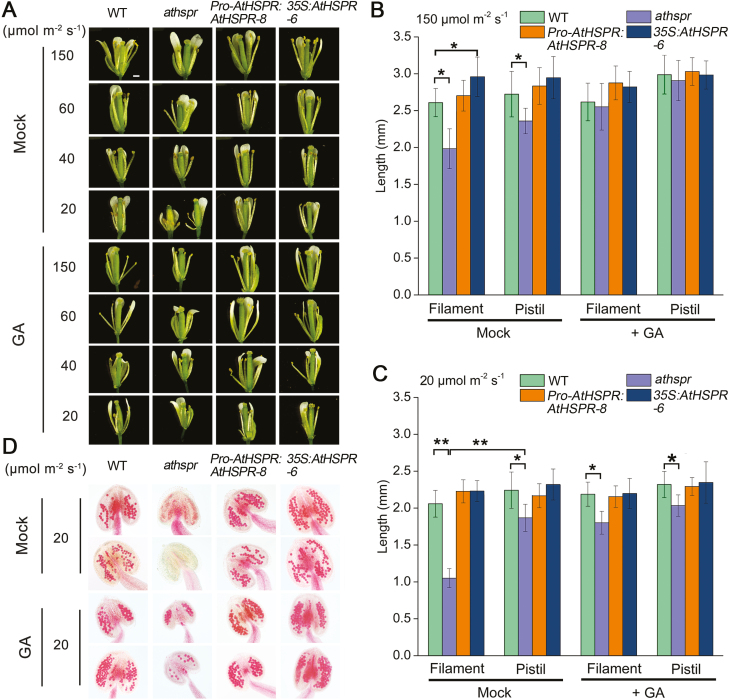
The Arabidopsis *Athspr* mutant displays defects in flower and stamen development under low-light and long-day conditions. Wild-type (WT), *Athspr*, *Pro-AtHSPR::AtHSPR-8*, and *35S::AtHSPR-6* plants were grown in long days (16/8 h light/dark) under different light intensities and sprayed twice a week until the onset of flowering with a solution either with or without 100 µM GA_3_. Spraying began when the plants were 21 d old. (A) Phenotypes of flowers at floral stage 13 under the different treatment conditions. The scale bar is 500 µm. (B, C) Filament and pistil lengths of the flowers with and without GA_3_ treatment for plants grown at (B) 150 µmol m^–2^ s^–1^ and (C) 20 µmol m^–2^ s^–1^. Data are means (±SD) of *n*=10 replicates. Significant differences between means were determined using Student’s *t*-test: *P<0.05, **P<0.01. (D) Alexander staining to show pollen vitality in flowers with and without GA_3_ treatment for plants grown at 20 µmol m^–2^ s^–1^. The scale bar is 50 µm. (This figure is available in color at JXB online.)

Compared with the WT at floral stage 13, the *Athspr* mutant exhibited obvious defects in flower development under a light intensity of 20 µmol m^–2^ s^–1^ (Fig. 7). While the WT, *Pro-AtHSPR::AtHSPR-8* and *35S::AtHSPR-6* lines maintained normal morphology under the different light conditions (Fig. 7A, B), ~75% of the *Athspr* flowers showed a globular or abnormal phenotype, in which their petals did not unfold normally (Supplementary Fig. S10A), their stamens were shriveled, and their pistils were abnormal (Fig. 7A, Supplementary Fig. S10B). Under 20 µmol m^–2^ s^–1^, the filament of *Athspr* flowers did not elongate (Fig. 7B, C), and the anthers did not dehisce to release viable pollen at floral stage 13.

To investigate whether *AtHSPR* was at least partially responsible for the reduced seed fertility under decreased light intensity, Alexander staining was used to assess the pollen viability at floral stage 12 under the range of different light intensities (Supplementary Fig. S10C, Fig. 7D). In plants grown at 150 µmol m^–2^ s^–1^ under LDs, the pollen viability of *Athspr* was similar to that of the WT, but as the light intensity decreased the number of normal pollen grains was reduced. Aborted anthers and shriveled pollen were noticeable when *Athspr* was grown under at 20 µmol m^–2^ s^–1^, indicating that the lack of seed set was partially due to defective development of the pollen. In contrast, the *Pro-AtHSPR::AtHSPR-8* and *35S::AtHSPR-6* lines showed a higher percentage of normal pollen grains compared to the WT. Application of GA was able to partially rescue both the defects in filament elongation (Fig. 7B, C) and the aborted anthers (Fig. 7D) in the *Athspr* mutant at 20 µmol m^–2^ s^–1^.

**Fig. 8.**
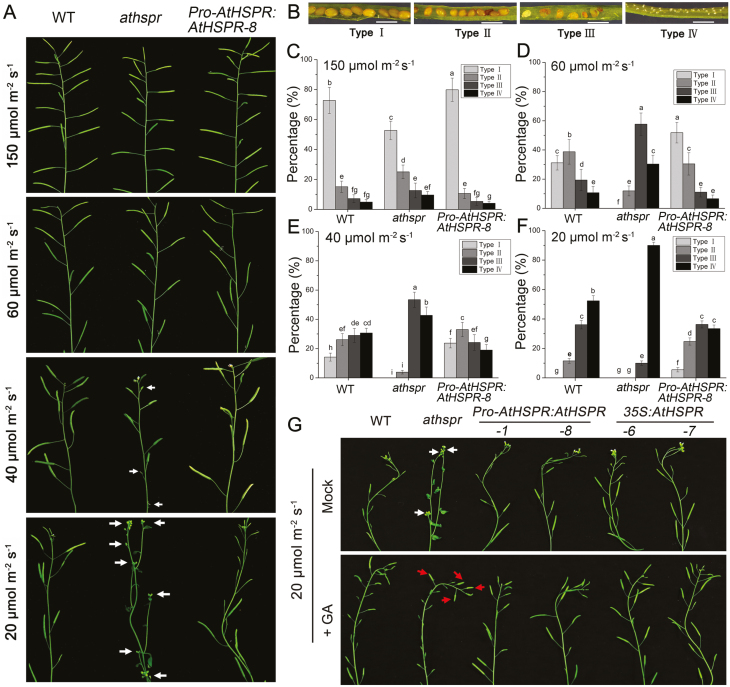
Mutation of the *AtHSPR* gene results in sterility under low-light and long-day conditions. (A) Silique phenotypes in primary stems of the Arabidopsis wild-type (WT), *Athspr* mutant, and *Pro-AtHSPR::AtHSPR-8* line grown under long days (16/8 h light/dark) and different light intensities. The arrows indicate unfilled or abnormal siliques in the *Athspr* mutant. (B) Siliques classified into four categories as detailed in Fig. 3C. Scale bars are 1 mm. (C–F) Percentage distribution of siliques within the four classification types for plants grown under the different light intensities. Data are means (±SD) of *n*=10 replicates. Different letters indicate significant differences as determined using ANOVA followed by Duncan’s multiple range test (*P*<0.05). (G) Main inflorescences in the WT, *Athspr* mutant, and *Pro-AtHSPR::AtHSPR-8* and *35S::AtHSPR* lines grown at 20 µmol m^–2^ s^–1^ with or without treatment with 100 µM GA_3_. Arrows in the Mock treatment indicate empty siliques in the *Athspr* mutant, whilst arrows in the +GA treatment indicate fertile siliques.

Under reduced light intensity, the siliques of WT, *Athspr*, and *Pro-AtHSPR::AtHSPR-8* plants were shorter and the number of sterile siliques was greater compared with high light (Fig. 8). Compared with the WT, *Athspr* plants exhibited a larger number of unfilled siliques when grown under a light intensity of less than 60 µmol m^–2^ s^–1^ (Fig. 8A–F). At 20 µmol m^–2^ s^–1^, over 95% of the *Athspr* siliques were empty or abnormal, while more than 30% were normal in the *Pro-AtHSPR::AtHSPR-8* plants (Fig. 8F). GA treatment of the *Athspr* mutant resulted in partial restoration of fertility at 20 µmol m^–2^ s^–1^ (Fig. 8G).

### AtHSPR positively regulates flowering-related genes and GA levels under low-light intensity

**Fig. 9.**
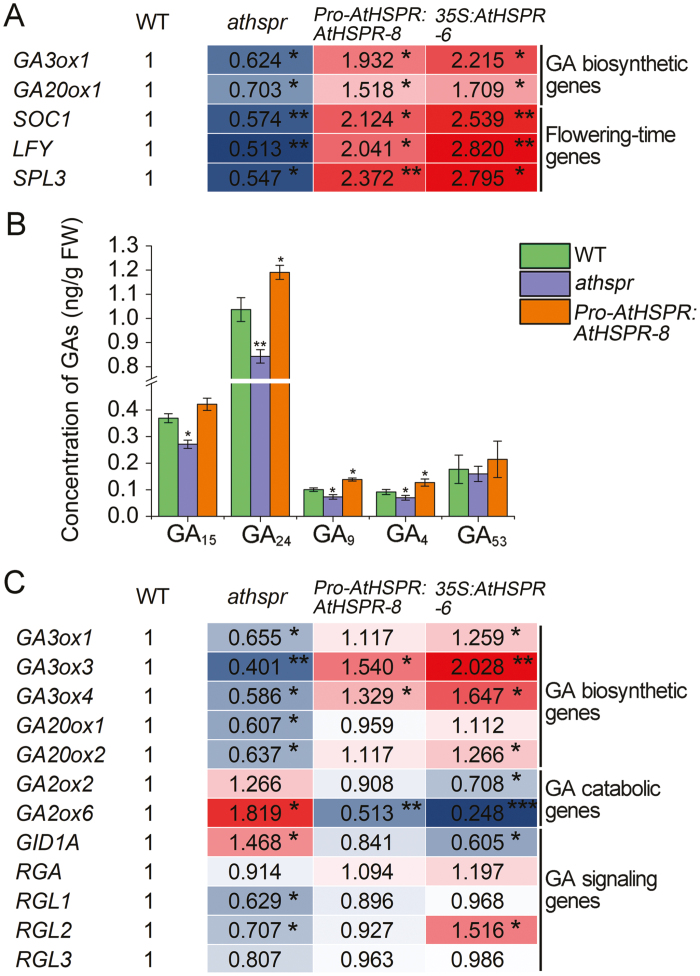
The expression levels of genes related to flowering time and GA biosynthesis, and concentrations of GAs in the Arabidopsis wild-type (WT), *Athspr* mutant, and *Pro-AtHSPR::AtHSPR-8* and *35S::AtHSPR-6* lines grown under low-light and long-day (16/8 h light/dark) conditions. (A) Relative expression of genes related to GA biosynthesis and flowering in under 40 μmol m^–2^ s^–1^, as determined by qRT-PCR. Total RNA was isolated from meristem tissues of 6-week-old plants. Expression levels are relative to those of the WT, which were set as 1. Data are means of *n*=3 replicates. (B) Endogenous concentrations of GAs in 7-week-old plants grown under 40 μmol m^–2^ s^–1^. Data are means (±SD) of *n*=3 replicates. (C) Relative expression of genes related to GA biosynthesis, GA catabolism, and GA signaling in flower buds from floral stage 13 of plants grown under 20 μmol m^–2^ s^–1^ as determined by qRT-PCR. Expression levels are relative to those of the WT, which were set as 1. Data are means of *n*=3 replicates. Significant differences compared with the WT were determined using Student’s *t*-test: *P<0.05, **P<0.01, ***P<0.001.

Plants of the WT, *Athspr*, *Pro-AtHSPR::AtHSPR-8*, and *35S::AtHSPR-6* were grown at 40 μmol m^–2^ s^–1^ and sampled at 6 weeks old for gene expression analysis. Compared with the WT, the relative expression levels of genes for GA biosynthesis (*GA3ox1* and *GA20ox1*) and flowering (*SOC1*, *LFY*, and *SPL3*) were significantly down-regulated before flowering in the *Athspr* mutant (Fig. 9A). In contrast, all of these genes showed higher expression levels in the *Pro-AtHSPR::AtHSPR-8* and *35S::AtHSPR-6* lines. We also quantified the concentrations of the main GA biosynthetic and catabolic intermediates in 7-week-old WT, *Athspr*, and *Pro-AtHSPR:AtHSPR-8* plants under the same light intensity (Fig. 9B). Consistent with the late-flowering and reduced seed-fertility phenotypes of the *Athspr* plants under low-light/LD conditions, we found that the levels of GA_4_ and the intermediates GA_15_, GA_24_, and GA_9_ were significantly reduced in the mutant compared with the WT.

We examined the expression levels of GA biosynthesis, catabolism, and signaling-related genes in the flower buds of the WT, *Athspr*, *Pro-AtHSPR::AtHSPR-8*, and *35S::AtHSPR-6* lines at 20 µmol m^–2^ s^–1^. At floral stage 13, the relative expression GA synthesis genes (*GA3ox1*, *GA3ox3*, *GA3ox4*, *GA20ox1*, and *GA20ox2*) was significantly down-regulated whilst catabolism genes (*GA2ox2* and *GA2ox6*) were up-regulated in the *Athspr* mutant compared with the WT (Fig. 9C). Conversely, the *Pro-AtHSPR::AtHSPR-8* and *35S::AtHSPR-6* lines showed higher expression levels of *GA3ox1*, *GA3ox3*, *GA3ox4*, and *GA20ox2* and lower expression levels of *GA2ox2* and *GA2ox6*. The expression of the GA receptor gene *GID1A* was up-regulated in *Athspr* compared to WT, whereas the expression of the GA signaling genes *RGL1/2* was down-regulated (Fig. 9C). In contrast, *GID1A* was down-regulated and *RGL2* was up-regulated in the flower buds of the *35S::AtHSPR-6* plants. Taken together, these results indicated that AtHSPR positively regulated light intensity-mediated control of both flowering time and fertility in a partially GA-dependent way.

### AtHSPR interacts with KNAT5 and OFP1 *in vitro*

Both the late-flowering and sterility phenotypes in *Athspr* under SD and low-light conditions were rescued by exogenous application of GA, suggesting that *AtHSPR* may act upstream of GA biosynthesis. Screening to determine whether AtHSPR had transcription-factor activity indicated that GAL4 DNA-binding domain (GAL4BD)-AtHSPR fusion proteins did not activate a GUS reporter gene in transient expression assays in Arabidopsis protoplasts (Supplementary Fig. S11). This implied that AtHSPR was not a canonical transcription factor.

**Fig. 10.**
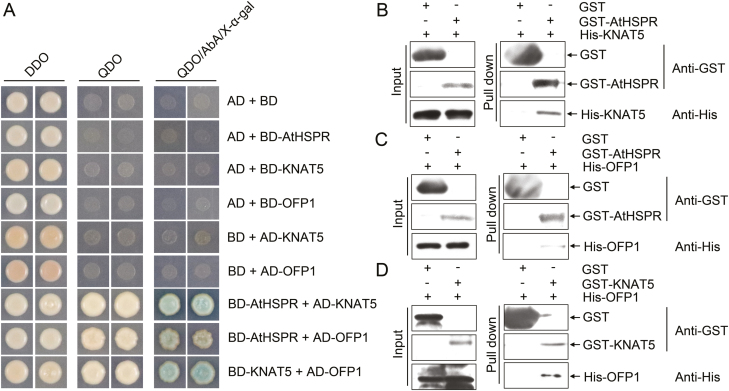
AtHSPR interacts with KNAT5 and OFP1 *in vitro*. (A) Interactions of AtHSPR with KNAT5 or OFP in yeast cells. Yeast cells were co-transformed with constructs encoding the binding domain (BD) fused to AtHSPR, KNAT5, and OFP1, and the activation domain (AD) fused to KNAT5 and OFP1, and then plated on selective double dropout medium (DDO, SD/–Leu/–Trp), quadruple dropout medium (QDO, SD/–Ade/–His/–Leu/–Trp), and QDO/AbA/X-α-gal (QDO supplemented with aureobasidin A and X-α-gal). (B–D) *In vitro* GST pull-down assays were performed by incubating GST-AtHSPR or GST-KNAT5 with His-KNAT5 or His-OFP1. GST served as the negative control. The experiments were repeated independently three times with similar results.

To examine the potential molecular function of AtHSPR in the GA pathway, we performed yeast two-hybrid assays. These demonstrated that AtHSPR interacted with two transcription factors, KNAT5 and OFP1 (Fig. 10A). The results also confirmed the interaction between KNAT5 and OFP1 in yeast cells, which was consistent with a previous study by Hackbusch *et al.* (2005). We then conducted pull-down assays to further test whether AtHSPR directly interact with KNAT5 and OFP1 *in vitro*, and found that histidine-tagged KNAT5 and OFP1 (His-KNAT5 and His-OFP1) could be pulled down by GST-tagged AtHSPR (GST-AtHSPR) (Fig. 10B, C). A similar result was obtained between GST-KNAT5 and His-OFP (Fig. 10D). These results indicated that AtHSPR directly interacted with KNAT5 and OFP1 *in vitro*. Previous studies have suggested that KNOX family proteins may mediate negative regulation of GA biosynthesis in the meristem (Sakamoto *et al.*, 2001; Chen *et al.*, 2004; Hackbusch *et al.*, 2005; Jasinski *et al.*, 2005). In addition, OFP1 has been identified as an important transcriptional repressor that directly controls the expression of *GA20ox1*, which encodes a key enzyme in the biosynthesis of GA (Hackbusch *et al.*, 2005; Wang *et al.*, 2007). Taken together, this suggests that the AtHSPR protein may interact with the transcription factors OFP1 and KNAT5 to regulate expression of genes related to GA homeostasis.

## Discussion

### AtHSPR acts as a positive regulator in flowering time and seed set

Our results indicated that AtHSPR acts as a positive regulator of flowering time and seed set. The *Athspr* mutant exhibited delayed transition to flowering under both LDs and SDs, with greater differences under the latter (Fig. 1). By contrast, plants over-expressing *AtHSPR* flowered earlier. The production of filled seed requires the formation of normal flowers capable of pollination, the production of viable pollen grains, and the healthy development of embryos (Liu *et al.*, 2017). The seed fertility in *Athspr* was markedly reduced under SDs and under low-light intensity, as was flower initiation (Fig. 3). This indicated that expression of *AtHSPR* is required for flowering and seed fertility in varying environmental conditions, and that the gene may provide an evolutionary advantage for reproductive success. In addition, transgenic lines expressing *AtHSPR* under the control of different promoters did not show significant differences in flowering time, even though the *35S::AtHSPR* lines showed a much higher expression level (Fig. 1, Supplementary Fig. S2). It is possible that a threshold level of functional AtHSPR protein was reached at a specific stage of development in either of these over-expression conditions, or that a co-factor (or co-factors) may have been limiting its function in the regulation of flowering.

AtHSPR/SMXL4 belongs to the SMXL family (Khosla and Nelson, 2016), members of which are involved in strigolactone (SL) signal transduction (Soundappan *et al.*, 2015; Wang *et al.*, 2015; Liang *et al.*, 2016). However, there are no reports of other members of the SMXL family being involved in the regulation of flowering time or seed set. In Arabidopsis seedlings, the signaling pathways initiated by GA and by the synthetic SL rac-GR24 converge at the level of transcription of a common gene set (Lantzouni *et al.*, 2017), which includes the gene encoding the GA catabolic enzyme GA2ox2. Further studies will be needed to determine whether the SMXL proteins associated with SL signaling are involved in the regulation of plant flowering and seed set and/or participate in the regulation of crosstalk between GA and SL signaling.

### AtHSPR regulates flowering and seed set in GA- and light intensity-dependent manners

Our phenotypic characterizations, physiological experiments, and gene expression assays strongly suggested that the *Athspr* mutant was defective in the GA pathway and hence that AtHSPR may regulate GA levels. Such regulation would influence the transcription of genes related to flowering time. Firstly, *Athspr* plants exhibited several phenotypes that were similar to many GA-defective mutants, including semi-dwarf growth, darker leaves, and decreased fertility (e.g. Figs 1, 3). These *Athspr* phenotypes were similar to those in the *ga1* mutant, which has significantly delayed flowering under LDs and fails to flower under SDs (Wilson *et al.*, 1992; Micheals and Amasino, 1999).

Secondly, the late flowering and reduced fertility in the *athspr* plants could be partially rescued by applying exogenous GA under SD and under low-light conditions (Figs 2, 3). Previous studies have shown that bioactive GAs coordinate with other hormone signals (e.g. jasmonate, auxin) to regulate stamen development at various floral developmental stages, including filament elongation, anther dehiscence, pollen maturation, and silique elongation (Cheng *et al.*, 2009; Plackett *et al.*, 2011; Song *et al.*, 2013). Normal, unfertilized pistils do not develop into siliques because of low levels of GAs. After fertilization, GA metabolism is normally activated, which in turn triggers GA signaling and silique development (Hu *et al.*, 2008; Rieu *et al.*, 2008; Dorcey *et al.*, 2009; Gomez *et al.*, 2016). Several GA-deficient Arabidopsis mutants, such as *ga1-3*, the *ga3ox1 ga3ox3* double-mutant, and the *ga3ox1 ga3ox3 ga3ox4* triple-mutant, are male-sterile, have short stamen filaments and poorly developed petals, and lack viable pollen (Cheng *et al.*, 2004; Hu *et al.*, 2008). Likewise, our results showed that mutation of *AtHSPR* caused a significant defect in fertility, repressed petal growth, shortened stamen filaments, and decreased the number of viable pollen grains under SD and under low-light conditions (Figs 3, 7, 8). Since these phenotypes are so similar to those seen in other GA-related mutants, they may be due to decreased levels of endogenous GAs in *Athspr*.

Thirdly, previous transcriptome data has shown that some GA metabolism and signaling genes are altered in the *Athspr* mutant under LDs (Supplementary Table S2). Our qRT-PCR analysis showed that the expression of GA biosynthetic, catabolic, and signaling genes was altered in the apical tissues of *Athspr* plants under SDs and in flower buds under low-light conditions (Figs 4A, 9C). These changes were positively correlated with the perturbation of the endogenous levels of GA biosynthetic and catabolic intermediates in the mutant (Figs 4C, 9B). Furthermore, the down-regulation of negative regulators of GA signaling (*RGL1*, *RGL2*, and *RGL3*) and up-regulation of the GA receptor *GID1A* in *Athspr* may have been due to feedback regulation in the GA pathway (Griffiths *et al.*, 2006). GA has been shown to act as a positive regulator that directly promotes the expression of floral integrator genes such as *SOC1* under SDs (Moon *et al.*, 2003). In the *Athspr* mutant, *SOC1* expression decreased in the meristem in response to SDs, which was associated with lower levels of bioactive GAs, whereas over-expression of *AtHSPR* enhanced the expression of *SOC1* (Fig. 9). Furthermore, depletion of GAs and reduction of GA signaling in the SAM reduces the expression of transcriptional regulators such as *SPL*s during floral induction under LDs (Galvão *et al.*, 2012; Porri *et al.*, 2012). The levels of *SPL3* and *SPL9* transcripts were reduced in the *Athspr* mutant and increased in *Pro-AtHSPR::AtHSPR-8* plants under SD and under low-light conditions, which could be due to the decreased levels of GAs in *Athspr* (Figs 4, 9). The observed functions of *AtHSPR* in the control of flowering time were most similar to those of other known genes such as *NO FLOWERING IN SHORT DAY* (*NFL*) and *SVP* that contribute to flowering regulation by altering the levels of GAs under SDs (Andrés *et al.*, 2014; Sharma *et al.*, 2016). In addition, we have previously demonstrated that mutation of *AtHSPR* reduces the ABA content under LDs (Yang *et al.*, 2015). ABA has been reported to activate ﬂowering under LDs but not under SDs in Arabidopsis (Riboni et al., 2013, 2016). This result is consistent with the late-flowering phenotype of the *Athspr* mutant in our present study, suggesting that the decreased ABA content may also partially contribute to the delayed flowering under LDs. Taken together, these results suggest that *AtHSPR* could function in the GA pathway and thus control flowering time by affecting levels of GA or GA intermediates and ABA.

Our results also revealed negative correlations between flowering time, pollen viability, seed set, and light intensity. The phenotypes of the *Athspr* mutant were more pronounced under lower light conditions, suggesting that *AtHSPR* is also involved in regulating flowering time in a light intensity-dependent manner. Previous studies have showed that changes in the quality and intensity of light affect flowering time (Cerdán and Chory, 2003; Kim *et al.*, 2008). *MYR1* and *MYR2* repress flowering under decreased light intensity partly due to their negative effects on levels of bioactive GAs (Zhao *et al.*, 2011). GAs also coordinate with light signals to regulate plant development (de Lucas *et al.*, 2008; Feng *et al.*, 2008; Li *et al.*, 2016). Our results indicated that *Athspr* was defective not only in the GA pathway, but also in the light intensity-dependent pathway.

**Fig. 11.**
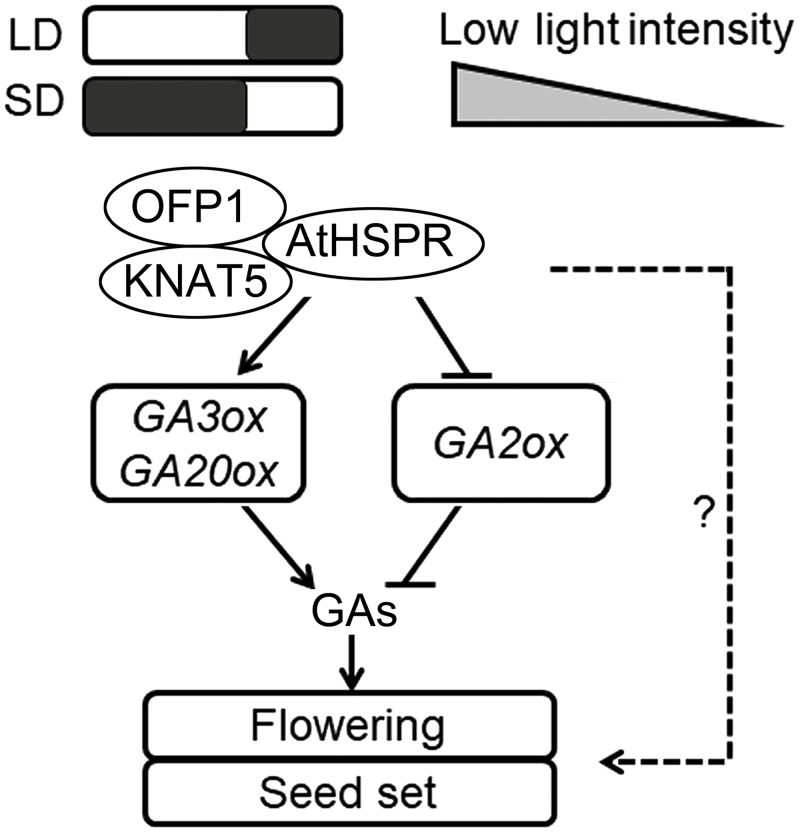
A simplified model showing the role of AtHSPR in GA- and light intensity-dependent regulation of flowering time and seed set in Arabidopsis. Under long-day (LD), short-day (SD), and low light-intensity conditions, AtHSPR may interact with OFP1 and KNAT5 and affect the expression of GA synthesis genes (*GA3ox* and *GA20ox*) and GA catabolism genes (*GA2ox*), which subsequently activates GA-dependent signaling. AtHSPR may also interact with the light intensity-dependent pathway to promote flowering time and seed set. Lines with arrows indicate promotion, lines with blocked ends indicate inhibition.

Yeast two-hybrid and GST pull-down assays confirmed that AtHSPR interacted directly with OFP1 and KNAT5 *in vitro* (Fig. 10). OFP1 in Arabidopsis plays a role in regulating flowering time (Zhang et al., 2016, 2018), and *GA20ox1* has been identified as a target gene of OFP1 (Wang *et al.*, 2007). In addition, KNOX family proteins have also been suggested to directly bind to the promoter region of *GA20ox1* and to repress transcription of *GA20ox1* (Sakamoto *et al.*, 2001; Chen *et al.*, 2004; Jasinski *et al.*, 2005), and because the nucleus-localized OFP1 protein lacks an apparent DNA-binding domain, it may regulate the expression of *GA20ox1* through interactions with BELL or KNOX transcription factors (Hackbusch *et al.*, 2005; Wang *et al.*, 2007). Taken together, AtHSPR can be considered as a co-factor and may potentially form a complex with OFP1 and KNAT5 in regulating the expression of *GA20ox1* or other GA-related target genes, and thereby contribute to GA-dependent regulation of flowering time and seed set (Fig. 11).

### Conclusions

Our results show that *AtHSPR* positively modulates flowering time and flower development, mainly via GA- and light intensity-dependent pathways, under LD, SD, and low-light conditions. However, the exact mechanism of AtHSPR action in GA-dependent regulation of development remains unclear, and whether it interacts with OFP1 or forms a complex with KNAT5-OFP1 needs to be further examined. Future characterization of AtHSPR will benefit from the construction of multiple mutants among *Athspr*, *ofp1*, and *knat5*, which will also help in our understanding of the functions of AtHSPR in promoting flowering and regulating seed set.

## Supplementary data

Supplementary data are available at *JXB* online.

Fig. S1. Complementation of the *Athspr* mutant with *Pro-AtHSPR::AtHSPR* rescues the defective phenotypes.

Fig. S2. Relative expression levels of *AtHSPR* in wild-type and transgenic over-expression lines under LDs.

Fig. S3. Mutation of *AtHSPR* affects floral transition.

Fig. S4. Mutation of *AtHSPR* affects reproductive organ development.

Fig. S5. Tissue-specific expression of GUS under the control of the *AtHSPR* promoter.

Fig. S6. Diurnal time-course of *AtHSPR* expression in response to LDs and SDs.

Fig. S7. Leaf angles of wild-type, *Athspr*, and *Pro-AtHSPR::AtHSPR-8* plants under LD and SD conditions.

Fig. S8. Responses of hypocotyl length in wild-type and *Athspr* seedlings when grown under red light, far-red light, or blue light.

Fig. S9. Responses of hypocotyl length in wild-type and *Athspr* seedlings when grown under low-light LDs, SDs, or dark conditions.

Fig. S10. Defects in flower and stamen development in the *Athspr* mutant grown under low-light intensity/LD conditions.

Fig. S11. The GAL4BD-AtHSPR fusion protein does not transcriptionally activate the GAL4 promoter.

Table S1. List of primer sequences used in this study.

Table S2. The expression levels of GA metabolism and signaling genes in the wild-type and *Athspr* mutant.

eraa128_suppl_Supplementary_Table_S1

eraa128_suppl_Supplementary_Table_S2

eraa128_suppl_Supplementary_Figures_S1_S11

## Abbreviations

AtHSPR: *Arabidopsis thaliana heat shock protein-related*
AP1: APETALA1
FT: FLOWERING LOCUS T
GA: gibberellin
GA20ox: GA 20-OXIDASE
GA3ox: GA 3-OXIDASE
GA2ox: GA 2-OXIDASE
GID1: GIBBERELLIC ACID INSENSITIVE DWARF 1
LD: long day
LFY: LEAFY
RGA: REPRESSOR OF GA1-3
RGL: REPRESSOR OF GA1-3-LIKE
SAM: shoot apical meristem
SD: short day
SMXL: SUPPRESSOR OF MORE AXILLARY GROWTH2 (MAX2)-LIKE
SOC1: SUPPRESSOR OF OVEREXPRESSION OF CONSTANS 1
SPL: SQUAMOSA PROMOTER BINGING PROTEIN-LIKE
OFP1: OVATE FAMILY PROTEIN1
KNAT5: KNOTTED1-LIKE HOMEOBOX5
