# Supplementary material for: AtHSPR is involved in GA- and light intensity-mediated control of flowering time and seed set in Arabidopsis
Source: J Exp Bot. 2020 Mar 10;71(12):3543–59. doi: 10.1093/jxb/eraa128 (PMC7475253; doi:10.1093/jxb/eraa128)
Supplement: eraa128_suppl_Supplementary_Table_S1 [file eraa128_suppl_supplementary_table_s1.pdf]

Table S1：Primer sequences used in experiments described in the text

	Genes	Forward	Reverse
	AtHSPR	CCAAAAGGATGAATTGAGTGGACTG	TTCTGCCCTCCAAGCAGTCATAC
	UBQ10	AAATCTCGTCTCTGTTATGCTTAAGAAG	TTTTACATGAAACGAAACATTGAACTT
	PP2A	TATCGGATGACGATTCTTCGTGCAG	GCTTGGTCGACTATCGGAATGAGAG
	GA20ox1	GCCTGTAAGAAGCACGGTTTCT	CTCGTGTATTCATGAGCGTCTGA
	GA20ox2	CCCAAGGCTTTCGTTGTCAA	CCGCTCTATGCAAACAGCTCT
	GA3ox1	TCCGAAGGTTTCACCATCACT	TCGCAGTAGTTGAGGTGATGTTG
	GA3ox3	TCCTACCCGGTTTGCC	ACGGTGCATTGTACTTC
	GA3ox4	GCCGATGACTCCTACC	ACACTTGTAGCCCTCC
	GA2ox2	CCCTCAAATTTTCCGTGAGT	CAGCATTTTACTCAGAGTGTC
	GA2ox6	GCGTTAAGTGGCGTTG	CTATGCCTCACGCTAGT
For qRT-PCR	*RGA*	CATTCCCGGAAACGCGATTTATCAG	TCACCGTCGTTCCTATGACTCCA
	RGL1	CAAGCTGGTGCTATGAGGAAAGTCG	GCGCAAACTTGAGATACGGACAAGA
	RGL2	CCGACCCGAATCTGAAACCTTAGTG	AAGCGCTTCGTTGAACCTATCGAG
	RGL3	CAAACGAAACCTCTAATCGCTGCAT	GGGCGAAATTGTCACAAAACGAAAC
	GID1a	GATGTCTTGATTGATCGCAGGAT	AGGAGGTTGCTCTTGATCTGCA
	SOC1	TGGGAGAAGGCATAGGAACATGC	CGCTTTCATGAGATCCCCACTTTTC
	LFY	ACGTGGCAAAAAGAACGGCTTAGA	CGCGTACCTGAATACTTGGTTCGTC
	AP1	CTCATAGCGTTCAAGTATCTTCTCC	GCTCATGAGATCTCTGTTCTCTGTG
	FLC	GCCAAGAAGACCGAACTCATGTTGA	CAACCGCCGATTTAAGGTGGCTA
	SPL3	CAACAATGCAGCAGGTTTCACG	TTTCCGCCTTCTCTCGTTGTG
	SPL9	TGTGGCTGGTATCGAACAGAGG	TTCCGGAAGCTGATGAAACCTG
Transcriptional activity assays	*GAL4BD-AtHSPR*	GGAGGCCGAATTCCCGGG ATGCGTACAGGGGCTTATACCGCG	TTGGCTGCAGGTCGACCTAATCAA CAAAGGAAACATGGAT

	Genes	Forward	Reverse
Yeast two-hybrid analyses	*BD-AtHSPR*	CATGGAGGCCGAATTCATGCGTACAGGG GCTTATACCGCG	GCAGGTCGACGGATCCCTAATCAACAAA GGAAACATGGAT
	BD-OFP1	CATGGAGGCCGAATTCATGGGTAATAACT ATCGGTTTAAGC	GCAGGTCGACGGATCCTTTGGAATGGGG TGGTGG
	BD-KNAT5	CATGGAGGCCGAATTCATGTCGTTTAACA GCTCCCAC	GCAGGTCGACGGATCCCGACTTCCCGGT CCGTTTA
	AD-OFP1	GGAGGCCAGTGAATTCATGGGTAATAACT ATCGGTTTAAGC	CGAGCTCGATGGATCCTTTGGAATGGGG TGGTGG
	AD-KNAT5	GGAGGCCAGTGAATTCATGTCGTTTAACA GCTCCCAC	CGAGCTCGATGGATCCCGACTTCCCGGT CCGTTTA
GST pull-down analysis	*pET30a-KNAT5*	GGCTGATATCGGATCCATGTCGTTTAACA GCTCCCAC	CCGCAAGCTTGTCGACCGACTTCCCGGT CCGTTTA
	pET30a-OFP1	GGCTGATATCGGATCCATGGGTAATAACT ATCGGTTTAAGC	CCGCAAGCTTGTCGACTTTGGAATGGGG TGGTGG
	pGEX-6P-1-AtHSPR	GGGGCCCCTGGGATCCATGCGTACAGG GGCTTATAC	GTCGACCCGGGAATTCTATCAACAAAGG AAACATGGA
	pGEX-6P-1-KNAT5	GGGGCCCCTGGGATCCATGTCGTTTAAC AGCTCCCAC	GTCGACCCGGGAATTCCGACTTCCCGGT CCGTTTA
