# Supplementary material for: AtHSPR is involved in GA- and light intensity-mediated control of flowering time and seed set in Arabidopsis
Source: J Exp Bot. 2020 Mar 10;71(12):3543–59. doi: 10.1093/jxb/eraa128 (PMC7475253; doi:10.1093/jxb/eraa128)
Supplement: eraa128_suppl_Supplementary_Table_S2 [file eraa128_suppl_supplementary_table_s2.pdf]

Table S2: The expression levels of GA metabolism and signaling genes in wild type and *athspr*

GA BIOSYNTHESIS	Gene Name	Log Fold Change (*athspr* / WT)	Up-Down-Regulation (*athspr* / WT)
AT4G02780.1	*CPS*	-0.691553854	Down
AT2G32440.1	*KAO2*	-0.485102976	Down
AT1G15550.1	*GA3ox1*	-0.103595101	Down
AT4G25420.1	*GA20ox1*	-0.299236431	Down
AT5G07200.1	*GA20ox3*	-0.710169532	Down
**GA CATABOLISM**			
AT1G30040.1	*GA2ox2*	0.982176504	Up
AT1G47990.1	*GA2ox4*	1.000323851	Up
AT1G02400.1	*GA2ox6*	1.069218932	Up
**GA SIGNALING**			
AT1G66350.1	*RGL1*	-0.24813209	Down
AT3G03450.1	*RGL2*	-1.138122919	Down
AT5G17490.1	*RGL3*	0.767877765	Up
AT2G45660.1	*SOC1*	-0.870984926	Down
AT2G33810.1	*SPL3*	-0.883756862	Down
AT2G42200.1	*SPL9*	-0.490456211	Down

GA-related genes differentially expressed in *athspr* mutant compared with wild-type C24 plants according to the RNA-seq experiments described previously (Yang *et al.* , 2015)
