# Supplementary material for: AtHSPR is involved in GA- and light intensity-mediated control of flowering time and seed set in Arabidopsis
Source: J Exp Bot. 2020 Mar 10;71(12):3543–59. doi: 10.1093/jxb/eraa128 (PMC7475253; doi:10.1093/jxb/eraa128)
Supplement: eraa128_suppl_Supplementary_Figures_S1_S11 [file eraa128_suppl_supplementary_figures_s1_s11.pdf]

Supplementary data

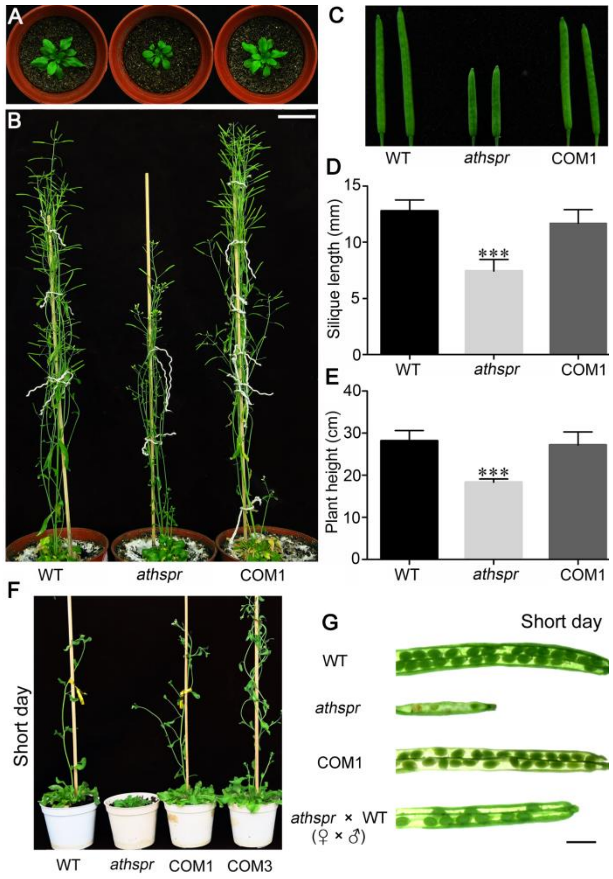

Fig. S1. Complementation of the *athspr* mutant with *Pro-AtHSPR::AtHSPR* rescued the defective phenotypes.

(A) The phenotypes of 28-day-old WT, *athspr* mutant and *AtHSPR* complementary (COM) plants under LD condition

(B)The phenotype and plant height of 10-week-old WT, *athspr* mutant and COM plants under LD condition.

(C-E) Silique length and plant height of 10-week-old WT, *athspr* and COM plants under LD condition. The asterisks above the bars indicate significant differences at $P < 0.001$ (***), based on Student's *t* test.

(F) Complementation of the *athspr* mutant with the *ProAtHSPR*:*AtHSPR* construct rescued the late-flowering phenotype under SD condition. Image taken at 10 weeks.

(G) The sterility phenotype of *athspr* mutant under SDs can be normally pollinated by WT pollen grains. Bar = 1 mm.

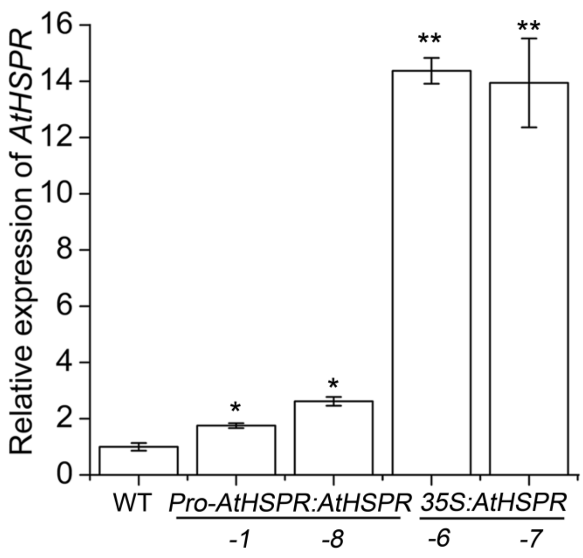

Fig. S2. Relative expression levels of *AtHSPR* in WT and transgenic over-expression lines under LDs.

Total RNA was isolated from the aerial part of 3-week-old plants. The asterisks above the bars indicate significant differences at $P < 0.05$ (*) or $P < 0.01$ (**), based on Student's *t* test.

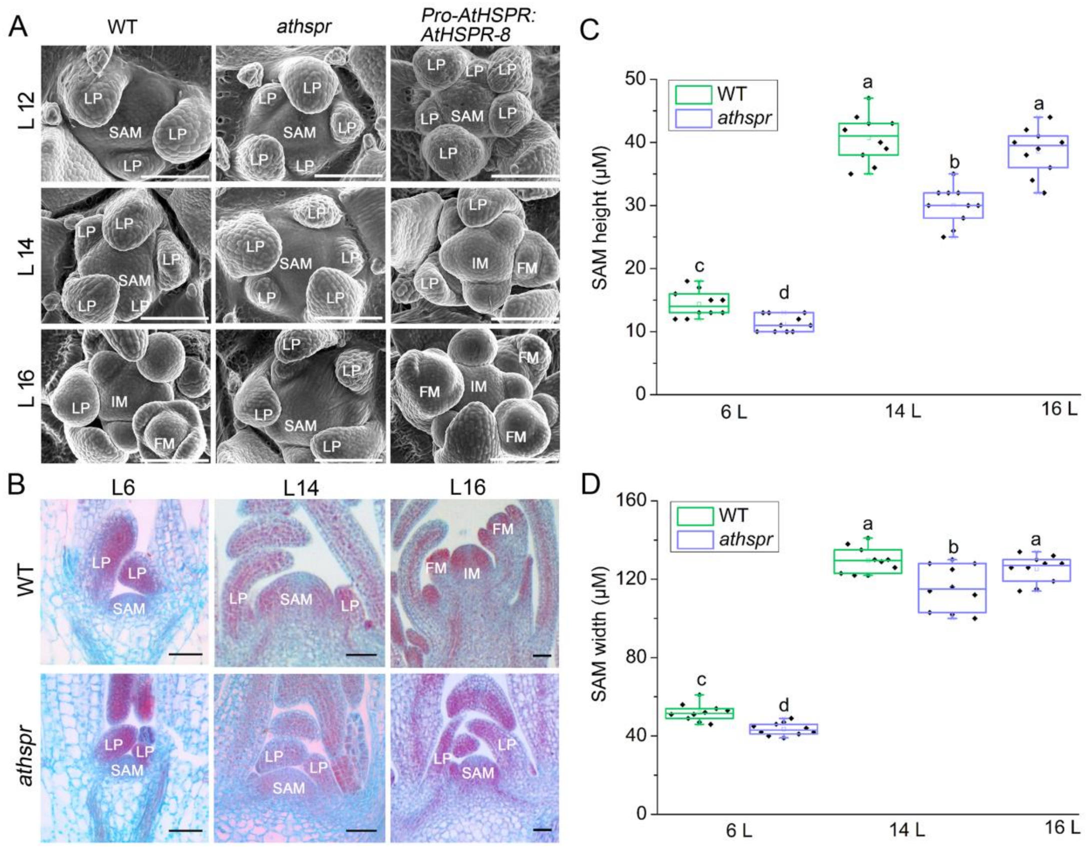

Fig. S3. Mutation of *AtHSPR* affects floral transition.

(A) SEM images of shoot apical meristem (SAM) or floral meristem (FM) in WT, *athspr* mutant, and *Pro-AtHSPR:AtHSPR-8* plants after producing 12, 14 or 16 leaves. Bar = 200 μm. LP, Leaf primordial, IM, Inflorescence shoot apical meristem.

(B) Paraffin sections of SAM in WT and *athspr* mutant plants after producing 6, 14 or 16 leaves. Bar = 50 μm.

(C-D) Quantitative analysis of SAM height (C) and width (D) in WT and *athspr* mutant plants after producing 6, 14 or 16 leaves. Data are shown as mean ± S.D. ($n = 10$ biologically independent samples). Different lowercase letters indicate significant differences as determined by ANOVA followed by Duncan's multiple range test ($P < 0.05$).

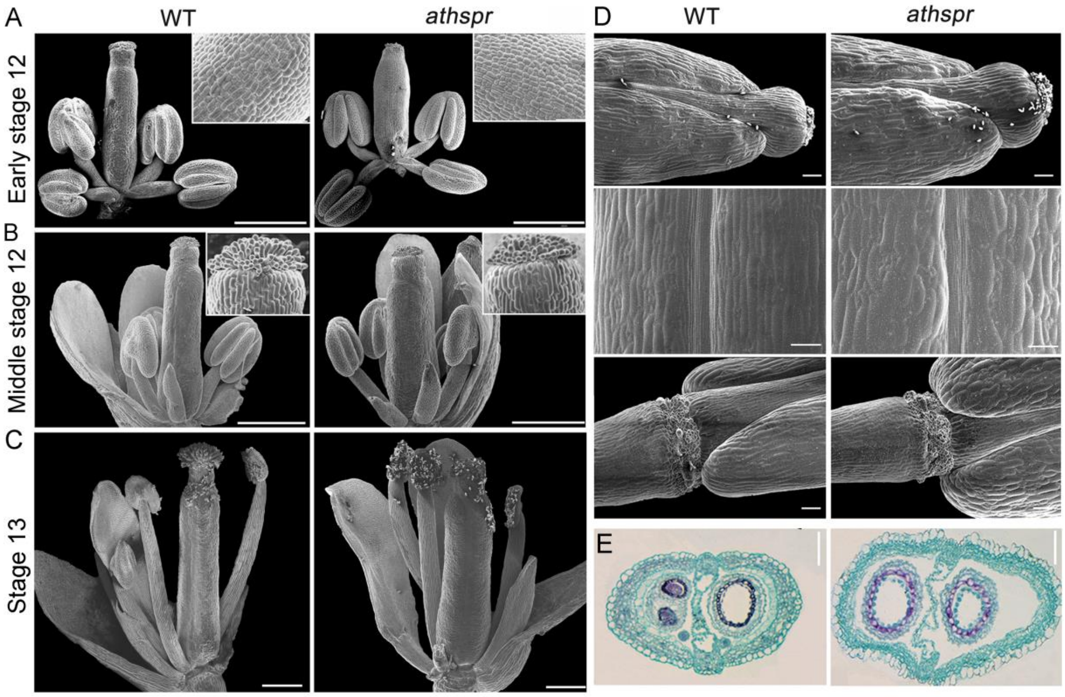

Fig. S4. Mutation of *AtHSPR* affects reproductive organ development.

(A-C) SEM images of flowers in WT and *athspr* mutant plants at early and middle floral stage 12 and floral stage 13 under LDs. Bar = 200 µm.

(D) SEM images of the apex (top panel), middle (middle) and base (lower) of siliques at stage 17 in WT and *athspr* mutant plants. Bar = 100 µm.

(E) Paraffin cross-section of stage 17 siliques in WT and *athspr* mutant. Bar = 100 µm.

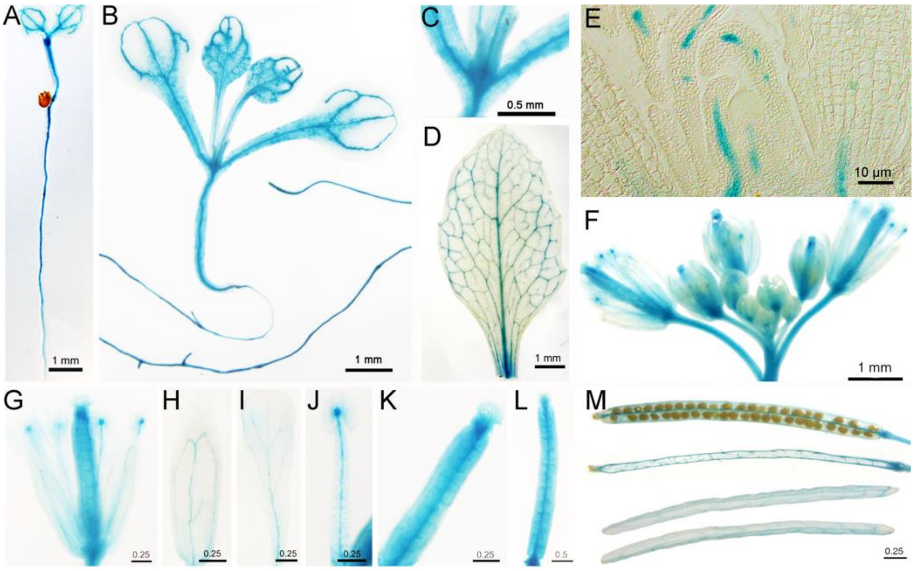

Fig. S5. Tissue-specific GUS expression from the *AtHSPR* promoter.

(A-D) Spatial visualization of transgenic plants expressing the *Pro-AtHSPR:GUS* construct in the C24 background by GUS staining in 7-day-old seedlings (A), 14-day-old seedlings (B, C) and 30-day-old mature leaf (D) grown under LDs. (C) is an enlargement of the apex shown in (B).

(E) GUS expression was analyzed in cross sections of shoot apex of 30-day-old *Pro-AtHSPR:GUS* plants.

(F-M) GUS staining for *AtHSPR* promoter analysis in inflorescences (F), flower (G), sepal (H), petal (I), filament and stamen (J), pistils (K), whole silique (L), and dissected silique (M). Scale bars = 1 mm in (A, B, D and F), 10 μm in (E), 0.25 mm in (G-K, and M), and 0.5 mm in (L).

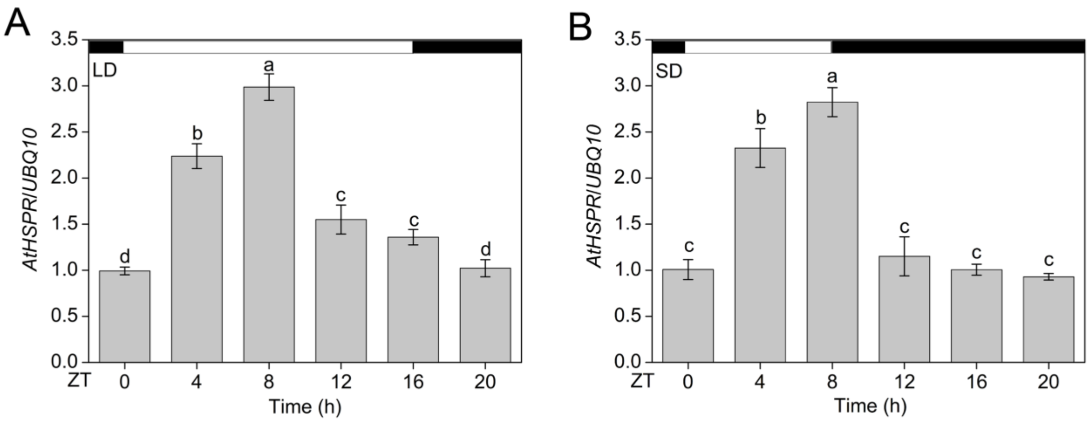

Fig. S6. Diurnal time course of *AtHSPR* expression in response to photoperiod.

Time course qRT-PCR analyses were performed using the aerial parts of 3-week-old wild-type plants grown under 16-h light/8-h dark (A) and 7-week-old wild-type plants grown under 8-h light/16-h dark (B). ZT=0 represents the time when the lights were switched on. *UBQ10* was used as the reference gene. White or black bars across the top represent light or darkness, respectively. Statistical significance was determined by a Duncan's multiple range test; significant differences ($P < 0.05$) are indicated by different lowercase letters.

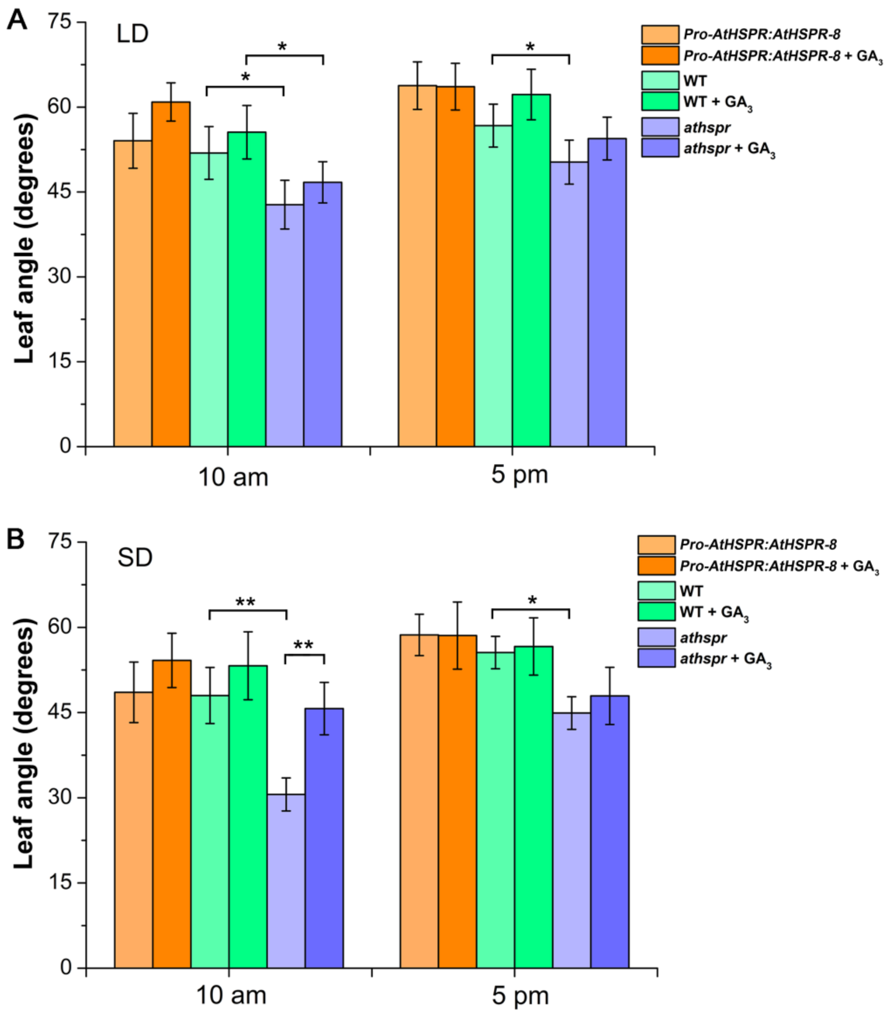

Fig. S7. Leaf angles of WT, *athspr* and *Pro-AtHSPR:AtHSPR-8* plants under LD and SD conditions.

(A-B) The leaf angles of 3-week-old plants grown under LD (A) or SD (B) conditions and treated with or without 100 µM GA_3 for 2 weeks were measured at 10 am or 5 pm.

The leaf angle between the petiole and the horizontal plane was measured for the most erect, fully expanded leaf on each plant. Values are means and standard deviation (n = 25). The asterisks above the bars indicate significant differences at $P < 0.05$ (*) or $P < 0.01$ (**), based on Student's t test.

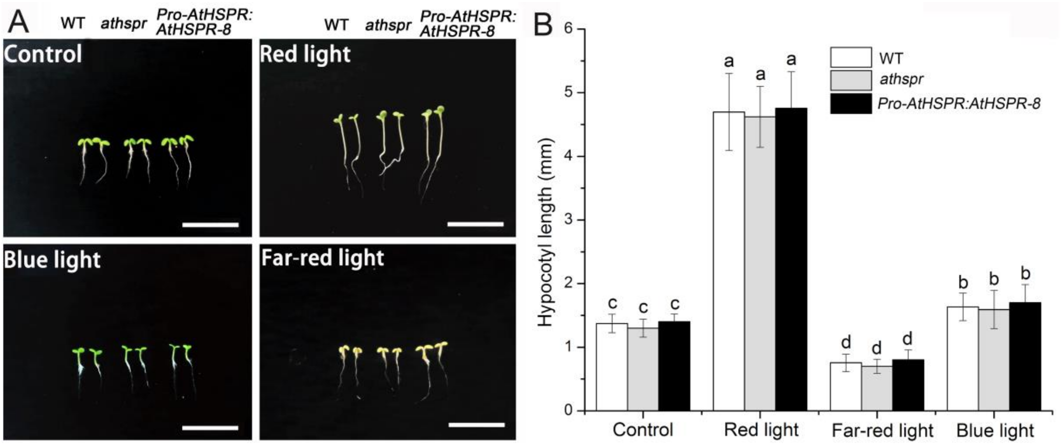

Fig. S8. *athspr* seedlings showed no significant difference compared to WT in hypocotyl length when grown under red light, far-red light, or blue light.

(A-B) The phenotype and hypocotyl length of seedlings of WT, *athspr* and *Pro-AtHSPR:AtHSPR-8* grown under red light, far-red light, and blue light conditions. Bar = 1 cm.

Statistical significance was determined by a Duncan's multiple range test (B), and significant differences ($P < 0.05$) are indicated by different lowercase letters.

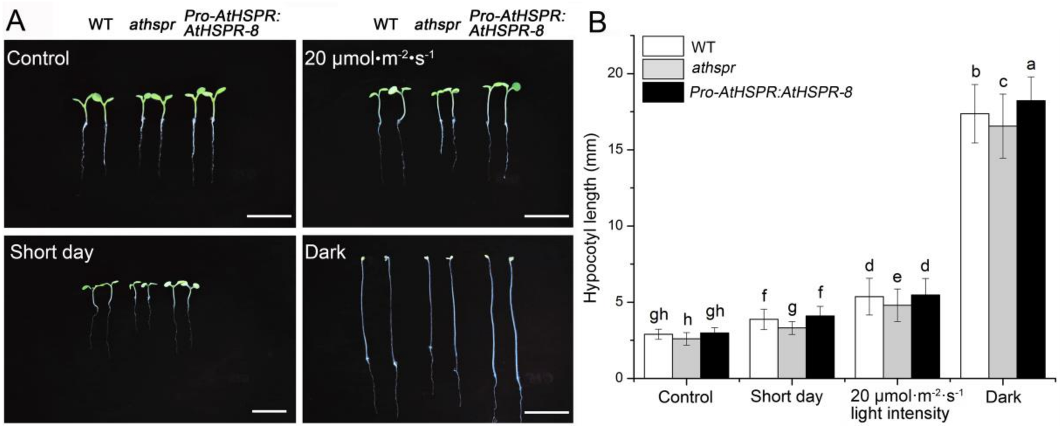

Fig. S9. *athspr* seedlings showed differences compared to WT in hypocotyl length when grown under low-light, SD, or dark conditions.

(A-B) The phenotype and hypocotyl length of seedlings of WT, *athspr* and *Pro-AtHSPR:AtHSPR-8* grown long days with under low-light (20 μmol m^{-2} s^{-1}), short days, and dark conditions. Bar = 1 cm.

Statistical significance was determined by a Duncan's multiple range test (B); significant differences (P < 0.05) are indicated by different lowercase letters.

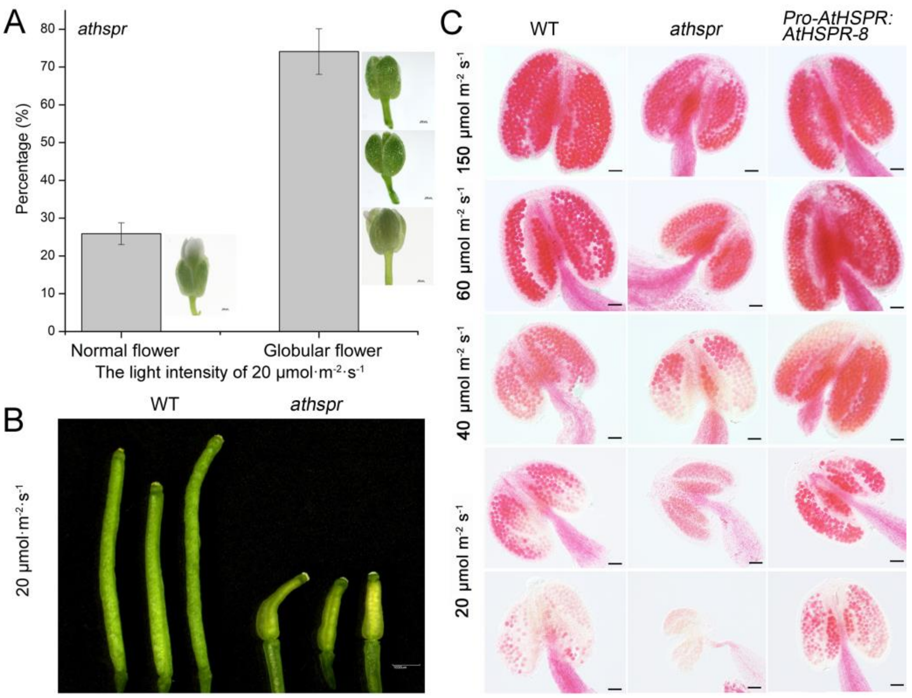

Fig. S10. The *athspr* mutant displayed defects in flower and stamen development under low-light intensity and LDs.

(A) The phenotype and percentage of normal flowers and globular flowers in *athspr* under 20 μmol m^{-2} s^{-1} light intensity. Three representative globular flowers are shown on right.

(B) The phenotype of pistils in WT and *athspr* mutant at floral stage 15. Scale bar = 1000 μm.

(C) Pollen vitality in WT, *athspr*, and *Pro-AtHSPR:AtHSPR-8* lines under low-light conditions. Bar = 50 μm.

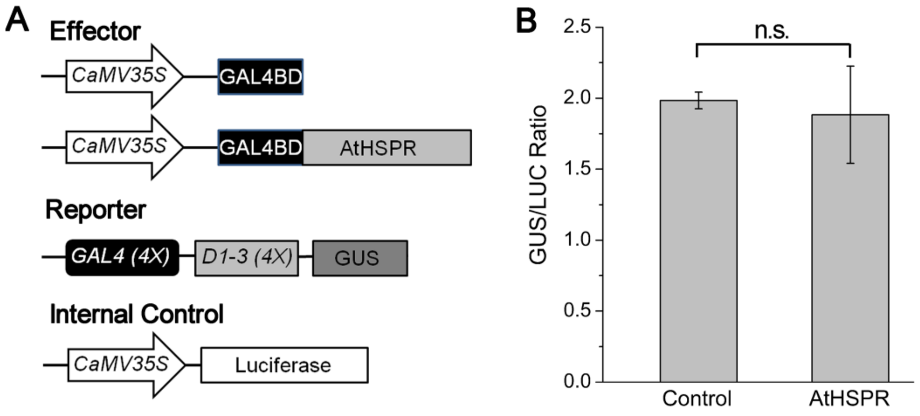

Fig. S11. The GAL4BD-AtHSPR fusion protein did not transcriptionally activate the GAL4 promoter.

(A) Schematic of the reporter and effectors used in the transient transactivation assays. AtHSPR-coding sequences were fused in-frame to the 3'-end of the GAL4 DNA-binding domain (GAL4BD)-coding sequence. The reporter and effector vectors were co-transformed into Arabidopsis protoplasts.

(B) Transient dual-luciferase reporter assays. Three measurements of GUS or REN activity were averaged and statistically treated using Student's *t*-test (n.s., no significant difference). Error bars indicate SD of the mean.
